# Bayesian nonparametric mixtures of categorical directed graphs for personalized causal inference

**DOI:** 10.1093/biostatistics/kxag026

**Published:** 2026-07-28

**Authors:** Federico Castelletti, Laura Ferrini

**Affiliations:** Department of Statistical Sciences, Università Cattolica del Sacro Cuore, Largo Gemelli, 1,Milan 20123, Italy; Department of Economics, Management and Statistics, Università degli Studi di Milano-Bicocca, Piazza dell’Ateneo Nuovo, 1, Milan 20126, Italy

**Keywords:** breast cancer, clustering, Dirichlet process prior, graphical model, personalized medicine

## Abstract

Quantifying the causal effect of a treatment on a disease is a crucial task in medical science for the administration of effective therapies. Typically, such causal effects are inferred from multivariate data that are collected on patients and recorded in the form of categorical variables, including risk factors involved in disease progression, treatment assignments, and disease status. This feature motivates an approach to causal inference based on categorical Directed Acyclic Graphs (DAGs), which provide an effective framework for causal reasoning in complex multivariate settings. In this context, traditional DAG-based methods assume population homogeneity and accordingly attribute a unique causal effect to all subjects. However, this assumption is often unrealistic in clinical contexts, since patients may exhibit heterogeneous characteristics, possibly linked to unmeasured features. To address this issue, we propose a Bayesian nonparametric methodology based on a Dirichlet Process mixture of categorical DAGs, which allows treatment effects to vary across individuals because of underlying clustering structures in the data. We develop a Markov chain Monte Carlo algorithm for Bayesian posterior inference and evaluate our methodology through simulation studies. We then analyze patients affected by HER2+ breast cancer undergoing therapies that may cause cardiotoxic side effects. Importantly, our findings show that approaches neglecting population heterogeneity may produce biased results, since they can over- or under-estimate the risk of cardiotoxicity across patients.

## 1. INTRODUCTION

Estimating cause-and-effect relations among variables is a pervasive issue in medical science, where a primary goal is to assess the effect of a therapy on the occurrence or progression of a disease. In modern biomedical studies, this problem is inherently multivariate as it involves multiple clinical, biological, and therapy-related measurements, which are frequently recorded in the form of categorical variables. As a consequence, to guarantee a coherent causal-effect estimation, one should account for possible complex interactions among all such variables. Graphical models based on Directed Acyclic Graphs (DAGs) provide a powerful framework for representing and learning dependence structures from multivariate categorical data. Crucially, DAGs enable a rigorous definition of causal effects on a target variable resulting from *hypothetical* interventions on other variables in the system. Moreover, under suitable assumptions on the data-generating process, causal effects can be identified and learned from observational data alone (Pearl 2000). A key issue addressed in this paper is data heterogeneity, which arises when causal mechanisms differ across individuals, typically due to the existence of latent subgroups. In biomedical applications, heterogeneity is particularly relevant, as patients often exhibit different clinical profiles, some of which may not be directly observed. In turn, causal-inference methods accounting for heterogeneity can provide a more reliable quantification of treatment effects, ultimately supporting personalized strategies for therapeutic administration; see Ma et al. (2015) for an overview.

As a motivating application, we consider a dataset of patients diagnosed with breast cancer and treated with different oncological therapies. In this context, the protein Human Epidermal growth factor Receptor 2 (HER2) has been identified as one of the main responsible of tumor progression and growth. Recent studies have shown that therapies targeting HER2 have a strong antitumor effect, improving the overall and progression-free survival (Slamon et al. 1987; Katzorke et al. 2013). However, they can cause cardiotoxicity as a side effect, with consequent heart failure and loss of left ventricular contractile function (Bowles et al. 2012; Dempke et al. 2023). Establishing the causal effect of anti-HER2 therapies on cardiotoxicity is therefore of key importance for the administration of appropriate anticancer treatments and to develop strategies for preventing and detecting cardiotoxicity in high-risk patients. Importantly also, there exist several factors, such as advanced age, hypertension, and arrhythmia, that predispose to cardiotoxicity (Lotrionte et al. 2013; Dempsey et al. 2021), and which should be included in the analysis.

In this paper, we introduce a novel Bayesian methodology based on an infinite mixture of categorical DAGs for causal discovery and causal effect estimation in the presence of heterogeneous data. Our model allows for the presence of latent subgroups of individuals/patients in the sample, each characterized by a possibly different causal structure and battery of related causal-effect parameters. Specifically, we assume that the multivariate distribution of the observables belongs to a Dirichlet Process (DP) mixture (Ferguson 1973) of categorical DAG models. Each mixture component reflects a factorization of the sampling distribution satisfying a set of conditional independencies imposed by the DAG. Under the latter, causal effects between variables are then defined according to do-calculus (Pearl 2000). Our methodology ultimately allows to quantify treatment effects of therapies w.r.t. the occurrence of cardiotoxicity at subject-specific level, thus leading to a more reliable decision process for the development of personalized strategies.

The literature on heterogeneous causal inference has grown extensively in the last years, particularly under the potential outcome framework (Rubin 2005). Assuming the existence of (latent) subgroups in the population, this issue is addressed through the definition and estimation of group-specific causal effects, known as Conditional Average Treatment Effects; see Dominici et al. (2021) for an overview. Stratification and heterogeneous causal inference have also been addressed using tree-based methods; see in particular Hahn et al. (2020) and Lee et al. (2021). These approaches use decision trees in order to partition individuals into groups that are homogeneous in terms of implied causal effects, based on the information provided by available covariates. Other approaches to heterogeneous causal inference using counterfactuals are based on Bayesian nonparametric methods; see, for instance, Linero and Antonelli (2022) and Zorzetto et al. (2024). In the potential outcome framework, covariates and risk factors are regarded as fixed quantities that one needs to adjust for when imputing the missing counterfactual. To account for heterogeneity, individuals are stratified and matched based on such confounders (e.g. age, gender, body mass index) before computing counterfactuals. However, when the causal clinical question involves various risk factors that may causally interact (e.g. smoking * → * arrythmia), a multivariate framework, such as based on DAGs, is more suitable for causal effect estimation; see also Dominici et al. (2021, Section 1.1) for related considerations.

Heterogeneous causal inference within Pearl’s framework has received relatively limited attention in the literature. A recent exception is Castelletti and Consonni (2023), who address heterogeneity in settings characterized by continuous (specifically, Gaussian) variables, with applications to genomic data. In contrast, our approach focuses on categorical measurements, which are particularly common in clinical research, whereas a crucial task is to establish the effect of a treatment status (corresponding to a binary variable) on the occurrence of a disease (a binary outcome); in addition, risk factors and patients’ characteristics are frequently recorded as categorical variables. All such features support the need of a methodology for causal inference based on a mixture of *categorical* DAGs.

Our approach indirectly allows for *model-based clustering* of multivariate categorical data. Existing methods for clustering categorical variables include both distance-based and model-based techniques, in particular the *k-modes* algorithm (Huang 1998) and the polytomous variable Latent Class Analysis method (Linzer and Lewis 2011), together with a few extensions. These methods could be used as alternatives in the first step of a two-stage procedure which stratifies observations into homogeneous groups, and then provides group-specific causal effect estimation. In this regard, simulation comparisons demonstrate a superior performance of our methodology, which is due to introduction of a graph-based model in the mixture-component kernels. This feature allows to capture data heterogeneity particularly when this is due to differences in the causal structures between clusters, rather than in the marginal distributions of variables only. As a final result, our unified approach can effectively characterize latent heterogeneity, leading to more reliable individual estimates of causal effects.

The rest of the paper is organized as follows. In Section 2, we provide some background material on DAGs and causal effects within a categorical modeling framework. In Section 3, we introduce our mixture model based on a DP prior, for which we detail the construction of the baseline mixing measure over the space of DAGs and allied parameters. We then describe a Markov Chain Monte Carlo (MCMC) strategy for posterior inference in Section 4. Section 5 is devoted to the analysis of breast cancer data and includes our causal-effect analysis to evaluate heterogeneous side effects of anti-HER2 therapies with respect to the occurrence of cardiotoxicity. We finally provide a discussion of our methodology in Section 6, together with possible future developments. Simulation studies and technical results, including the computation of prior and posterior predictive distributions required by our posterior sampler, are reported in the Supplementary Material.

## 2. BACKGROUND

### 2.1. Categorical DAG models

Consider a DAG $ \mathcal{D}=(V, E) $ with set of nodes $ V=\{1 , \dots, q\} $ and set of directed edges $ E\subseteq V\times V $. If * (u, v)∈ E *, we say that * u * is a *parent* of * v * and let $ \mathrm{pa}_{\mathcal{D}}(v) $ be the set of all parents of * v * in $ \mathcal{D} $. Moreover, we let $ \mathrm{fa}_{\mathcal{D}}(v):=v\cup\mathrm{pa}_{\mathcal{D}}(v) $ be the *family* of node * v * in the DAG. Consider now a collection of random variables $ X=(X_{1},\dots, X_{q}) $, such as clinical features that can be measured on patients and binary categorical variables indicating the administration of a therapy and the absence/presence of a disease. In the following, we assume that each $ X_{j} $, * j∈ V *, is categorical with set of levels $ \mathcal{X}_{j} $ and let $ x_{j}\in\mathcal{X}_{j} $ be one of its levels. Accordingly, $ X\in\mathcal{X}:=\times_{j\in V}\mathcal{X}_{j} $, whose generic element is $ x\in\mathcal{X} $. In addition, if for any * S⊂ V * we let $ X_{S}=(X_{j},j\in S) $, then $ X_{S}\in\mathcal{X}:=\times_{j\in S}\mathcal{X}_{j} $, with $ x_{S}\in\mathcal{X}_{S} $. Under $ \mathcal{D} $, the joint probability $ p(x)=\mathrm{Pr}(X_{1}=x_{1},\dots, X_{q}=x_{q}) $ admits the factorization


(2.1) \begin{align*} p(x)=\prod\limits_{j=1}^{q}\Pr(X_{j}=x_{j}\,|\, X_{\mathrm{pa}(j)}=x_{\mathrm{pa}(j)}),\end{align*}

which is also named the *observational distribution*. For the remainder of this section, we omit DAG $ \mathcal{D} $ from our notation and reason conditionally on a fixed DAG. Let now $ \theta_{s}^{S}=\Pr(X_{S}=s) $, $ s\in\mathcal{X}_{S} $, be a marginal probability for variables in * S⊆ V *. Moreover, let $ \theta^{j\,|\,\mathrm{pa}(j)}_{m\,|\, s}=\Pr(X_{j}=m\,|\, X_{\mathrm{pa}(j)}=s) $ be a conditional probability for $ X_{j} $ given configuration (level) * s * of $ X_{\mathrm{pa}(j)} $, with $ m\in\mathcal{X}_{j},s\in\mathcal{X}_{\mathrm{pa}(j)} $. Consider * n * observations from * X *, $ \boldsymbol{x}^{(1)},\dots , \boldsymbol{x}^{(n)} $, where each $ \boldsymbol{x}^{(i)}=(x^{(i)}_{1},\dots, x^{(i)}_{q})^{\top} $, and $ \boldsymbol{x}^{(i)}\in\mathcal{X} $, $ i\,=\,1 , \dots, n $. Also, let $ \boldsymbol{x}_{S}^{(i)} $ be the sub-vector of $ \boldsymbol{x}^{(i)} $ with components indexed by * S⊂ V *. If we collect the $ \boldsymbol{x}^{(i)} $’s into an * (n, q) * data matrix $ \boldsymbol{X} $, then the likelihood function can be written as


(2.2) \begin{align*}p(\boldsymbol{X}\,|\,\boldsymbol{\theta})&=\,\prod\limits_{i=1}^{n}\left\{\prod\limits_{x\in\mathcal{X}}\left\{p(\boldsymbol{x}^{(i)}\,|\,\boldsymbol{\theta})\right\}^{\mathds{1}\{\boldsymbol{x}^{(i)}=x\}}\right\}\nonumber\\ &=\,\prod\limits_{j=1}^{q}\left\{\prod\limits_{s\in\mathcal{X}_{\mathrm{pa}(j)}}\left\{\prod\limits_{m\in\mathcal{X}_{j}}\left\{\theta^{j\,|\,\mathrm{pa}(j)}_{m\,|\, s}\right\}^{n^{\mathrm{fa}(j)}_{(m, s)}}\right\}\right\},\end{align*}

now emphasizing the dependence on the DAG-parameter $ \boldsymbol{\theta} $ (corresponding to the collection of conditional probabilities in the equation) and where $ n^{\mathrm{fa}(j)}_{(m, s)}=\sum_{i\,=\,1}^{n}\mathds{1}\left\{\boldsymbol{x}^{(i)}_{\mathrm{fa}(j)}=(m, s)\right\} $ is the number of observations for which $ X_{\mathrm{fa}(j)}=(m, s) $. See also Castelletti et al. (2024) for further notation on categorical DAG models.

### 2.2. Causal effects for categorical DAGs

For a given collection of random variables whose multivariate distribution factorizes according to a DAG, we now focus on the *causal effect* of an intervention on $ X_{h} $, * h∈ V *, on a response variable of interest, say $ X_{j}:=Y $, * j≠q h *. In practice, such an intervention corresponds to assigning a treatment to an individual, equivalently fixing $ X_{h}=\tilde{x} $, where $ X_{h} $ is typically an *exposure* of * Y *, and this action can be denoted using Pearl’s do-operator $ \mathrm{do}(X_{h}=\tilde{x}) $ (Pearl 2003). We introduce three typical assumptions that are needed to properly perform causal inference in a DAG-based framework given observational data.

Assumption 1 (Structural causal model). In the observational distribution (2.1), each component $ p(X_{j}=x_{j}\,|\, X_{\mathrm{pa}(j)}=x_{\mathrm{pa}(j)}) $ corresponds to an autonomous and stable mechanism.

Assumption 2 (Causal sufficiency). The collection of variables $ (X_{1},\dots, X_{q}) $ does not include latent unmeasured confounders.

Assumption 3 (Faithfulness). The all and only conditional independencies embodied in the joint distribution of $ (X_{1},\dots, X_{q}) $ can be read-off from the DAG using the Markov property.

While Assumption 3 is meaningful when considering DAG model uncertainty (Section 3), Assumptions 1 and 2 imply the *postintervention* distribution


(2.3) \begin{align*} p(x\,|\,\mathrm{do}(X_{h}=\tilde{x}))=\left\{\begin{array}{@{}ll}\prod\limits_{j\neq h}p\big(X_{j}=x_{j}\,|\, X_{\mathrm{pa}(j)}=x_{\mathrm{pa}(j)}\big)&\textrm{if}\ X_{h}=\tilde{x}\\ 0&\textrm{otherwise},\end{array}\right.\end{align*}

where importantly all components * j≠q h * in the product are the same as in (2.1) because of Assumption 1. Assuming now for simplicity that both $ X_{h} $ and * Y * are binary variables with levels in $ \{0,1\} $, the causal effect of $ \mathrm{do}\{X_{h}=\tilde{x}\} $ on * Y * can be defined as


(2.4) \begin{align*} c_{y, h}:=\mathbb{E}\big[Y\,|\,\mathrm{do}(X_{h}=1)\big]-\mathbb{E}\big[Y\, |\,\mathrm{do}(X_{h}=0)\big];\end{align*}

see Pearl (2003). From a biomedical perspective, when * Y * denotes a disease status and $ X_{h} $ represents a treatment assignment, $ c_{y, h} $ can be interpreted as the average change in the disease probability consequent to treatment administration.

More in general, if $ X_{h} $ is polytomous with levels labeled as $ \{0,1 , \dots, L\} $, one can define a battery of causal effects by considering $ X_{h}=l $, for each $ l\,=\,1 , \dots, L $ in the first expectation of (2.4). In addition, an overall summary of the resulting collection can be defined and computed; see for instance Kvisgaard and Pensar (2024). According to the definition above, $ c_{y, h} $ involves a (marginal) postintervention distribution of * Y *. However, because of (2.3), the latter can be expressed in terms of observational distributions, simply by conditioning and then marginalizing w.r.t. a *valid adjustment set* * Z⊂ X * (Pearl 2003). A common choice for such an adjustment set is $ Z\,=\,X_{\mathrm{pa}(h)} $, namely the parents of $ X_{h} $, leading to


(2.5) \begin{align*}c_{y, h}&=\sum\limits_{s\in\mathcal{X}_{\mathrm{pa}(h)}}\mathbb{E}\big(Y\,|X_{h}=1, X_{\mathrm{pa}(h)}=s\big)\Pr\big(X_{\mathrm{pa}(h)}=s\big)\nonumber\\ &\quad-\sum\limits_{s\in\mathcal{X}_{\mathrm{pa}(h)}}\mathbb{E}\big(Y\,|\, X_{h}=0, X_{\mathrm{pa}(h)}=s\big)\Pr\big(X_{\mathrm{pa}(h)}=s\big);\end{align*}

see in particular Theorem 3.2.3 in Pearl (2000). Finally, under model (2.2), the causal effect in (2.5) can be expressed as a function of the DAG parameter $ \boldsymbol{\theta} $ as


(2.6) \begin{align*}\gamma_{y, h}(\boldsymbol{\theta})=\sum\limits_{s\in\mathcal{X}_{\mathrm{pa}(h)}}\left\{\left(\theta^{Y\,|\,\mathrm{fa}(h)}_{1\,|\,(1, s)}-\theta^{Y\,|\,\mathrm{fa}(h)}_{1|(0, s)}\right)\theta^{\mathrm{pa}(h)}_{s}\right\}.\end{align*}

## 3. DP MIXTURE OF CATEGORICAL DAG MODELS

In this section, we introduce our DP mixture of categorical DAG models. This can be written using the following hierarchical structure


(3.7) \begin{align*}\boldsymbol{x}^{(i)}\,|\,\boldsymbol{\theta}_{i},\mathcal{D}_{i}&\sim p(\boldsymbol{x}^{(i)}\,|\,\boldsymbol{\theta}_{i},\mathcal{D}_{i})\nonumber\\ (\boldsymbol{\theta}_{i},\mathcal{D}_{i})\,|\, H&\sim H\nonumber\\ H&\sim DP(M_{0},\alpha)\end{align*}

where $ DP(M_{0},\alpha) $ denotes a DP prior with baseline $ M_{0} $ and concentration parameter * α * (Ferguson 1973), and we now emphasize the dependence on DAG $ \mathcal{D}_{i} $ in the sampling distribution $ p(\boldsymbol{x}^{(i)}\,|\,\boldsymbol{\theta}_{i},\mathcal{D}_{i}) $.

A property of model (3.7) is that it induces a partition of the observations $ \boldsymbol{x}^{(1)},\dots , \boldsymbol{x}^{(n)} $ into clusters, with individuals assigned to the same cluster sharing the same DAG $ \mathcal{D} $ and DAG parameter $ \boldsymbol{\theta} $. Being a function of $ \boldsymbol{\theta} $, the causal effect * γ * will vary across clusters, so that our model is able to capture heterogeneous causal effects which are implied by the resulting clustering structure in the data. In addition, the expected number of clusters is controlled by * α *: each observation $ \boldsymbol{x}^{(i)} $ is associated with its own $ (\boldsymbol{\theta}_{i},\mathcal{D}_{i}) $-parameter as $ \alpha\rightarrow\infty $; on the contrary, if $ \alpha\rightarrow 0 $, then all observations are assigned to the same cluster, leading to a standard categorical DAG model (Castelletti et al. 2024); see also Müller and Rodriguez (2013) for related properties of the DP prior. Let now $ (\boldsymbol{\theta}^{\star}_{1},\mathcal{D}^{\star}_{1}),\dots,(\boldsymbol{\theta}^{\star}_{K},\mathcal{D}^{\star}_{K}) $ be the * K * unique (paired) values among $ (\boldsymbol{\theta}_{1},\mathcal{D}_{1}),\dots,(\boldsymbol{\theta}_{n},\mathcal{D}_{n}) $, and $ \{\xi_{i}\}_{i\,=\,1}^{n} $ a sequence of cluster-indicator variables such that $ \xi_{i}\in\{1 , \dots, K\} $ and $ (\boldsymbol{\theta}_{i},\mathcal{D}_{i})=(\boldsymbol{\theta}^{\star}_{\xi_{i}},\mathcal{D}^{\star}_{\xi_{i}}) $. Conditionally on $ \{\xi_{i}\}_{i\,=\,1}^{n} $, observations are i.i.d. within each cluster, so that the likelihood can be written as


(3.8) \begin{align*}p\left(\boldsymbol{X}\,|\,\{\xi_{i}\}_{i=1}^{n},\{\boldsymbol{\theta}_{i}\}_{i=1}^{n},\{\mathcal{D}_{i}\}_{i=1}^{n}\right)&=\,\prod\limits_{k=1}^{K}\left\{\prod\limits_{i : \xi_{i}=k}p\left(\boldsymbol{x}^{(i)}\,|\,\boldsymbol{\theta}^{\star}_{\xi_{i}},\mathcal{D}^{\star}_{\xi_{i}}\right)\right\}\nonumber\\ &=\,\prod\limits_{k=1}^{K}p\big(\boldsymbol{X}^{(k)}\,|\,\boldsymbol{\theta}^{\star}_{k},\mathcal{D}^{\star}_{k}\big),\end{align*}

with $ p\big(\boldsymbol{X}^{(k)}\,|\,\boldsymbol{\theta}^{\star}_{k},\mathcal{D}^{\star}_{k}\big) $ as in (2.2), and where $ \boldsymbol{X}^{(k)} $ is the $ (n_{k},q) $ matrix collecting all observations $ \boldsymbol{x}^{(i)} $ such that $ \xi_{i}=k $. Additionally, a generic count involved in the * k *-th component above will be denoted as $ {}_{k}n^{\mathrm{fa}(j)}_{(m, s)}=\sum_{i : \xi_{i}=k}\mathds{1}\big\{\boldsymbol{x}^{(i)}_{\mathrm{fa}(j)}=(m, s)\big\} $, which corresponds to the number of observations in cluster * k * for which the level taken by variables $ X_{\mathrm{fa}(j)} $ is equal to * (m, s) *.

Following Escobar and West (1995), we assign $ \alpha\sim\mathrm{Gamma}(c, d) $, while in the next sections we detail the construction of the baseline $ M_{0} $. This is structured as $ M_{0}=p(\boldsymbol{\theta}\,|\,\mathcal{D})p(\mathcal{D}) $, where the first term corresponds to a prior on the DAG parameter $ \boldsymbol{\theta} $ conditionally on DAG $ \mathcal{D} $, while the latter is a marginal prior over DAGs.

### 3.1. Baseline on DAG parameter

Conditionally on $ \mathcal{D} $, we first assign the prior $ p(\boldsymbol{\theta}\,|\,\mathcal{D}) $. To this end, consider for each node $ j\in\{1 , \dots, q\} $ and $ s\in\mathcal{X}_{\mathrm{pa}(j)} $ the parameter $ \big(\theta^{j\,|\,\mathrm{pa}(j)}_{m\,|\, s},m\in\mathcal{X}_{j}\big):=\boldsymbol{\theta}^{j\,|\,\mathrm{pa}(j)}_{s} $ corresponding to a $ |\mathcal{X}_{j}| $-dimensional vector collecting conditional probabilities for variable $ X_{j} $, given a configuration * s * of its parents $ X_{\mathrm{pa}(j)} $. We assign to each $ \boldsymbol{\theta}^{\, j\,|\,\mathrm{pa}(j)}_{s} $ a Dirichlet prior with hyperparameter $ \boldsymbol{a}^{\, j\,|\,\mathrm{pa}(j)}_{s}=\big(a^{j\,|\,\mathrm{pa}(j)}_{m\, |\, s},m\in\mathcal{X}_{j}\big) $, written as $ \boldsymbol{\theta}^{j\,|\,\mathrm{pa}(j)}_{s}\sim\mathrm{Dirichlet}(\boldsymbol {a}^{j\,|\,\mathrm{pa}(j)}_{s}) $, whose p.d.f. is


(3.9) \begin{align*}p\big(\boldsymbol{\theta}^{j\,|\,\mathrm{pa}(j)}_{s}\big{)}&=h\big(\boldsymbol{a}^{j\,|\,\mathrm{pa}(j)}_{s}\big)\prod\limits_{m\in\mathcal {X}_{j}}\left\{\theta^{j\,|\,\mathrm{pa}(j)}_{m\,|\, s}\right\}^{a^{j\,|\,\mathrm{pa}(j)}_{m\,|\, s}-1},\end{align*}

and $ h\big(\boldsymbol{a}^{j\,|\,\mathrm{pa}(j)}_{s}\big) $ is the prior normalizing constant. Let now $ \boldsymbol{\theta}^{j\,|\,\mathrm{pa}(j)}=\big(\boldsymbol{\theta}^{j\,|\,\mathrm{pa}(j)}_{s},s\in\mathcal{X}_{\mathrm{pa}(j)}\big) $. By assuming global and local parameter independence (Geiger and Heckerman 1997), respectively $ \perp\kern-1.7pt\kern-1.7pt\kern-1.7pt\perp_{j}\boldsymbol{\theta}^{j\,|\,\mathrm{pa}(j)} $ and $ \perp\kern-1.7pt\kern-1.7pt\kern-1.7pt\perp_{s}\boldsymbol{\theta}^{j\,|\,\mathrm{pa}(j)}_{s} $, a joint prior on $ \boldsymbol{\theta}=\big\{\boldsymbol{\theta}^{j\,|\,\mathrm{pa}(j)},j\in V\big\} $ can be written as


(3.10) \begin{align*}p(\boldsymbol{\theta})=\prod\limits_{j=1}^{q}\left\{\prod\limits_{s\in{\mathcal{X}}_{\mathrm{pa}(j)}}p\big(\boldsymbol{\theta}^{j\,|\,\mathrm{pa}(j)}_{s}\big)\right\}.\end{align*}

In what follows we implement the default choice $ a^{j\,|\,\mathrm{pa}(j)}_{m\,|\, s}=a/|{\mathcal{X}}_{\mathrm{fa}(j)}| $, $ a\,\gt\,0 $, leading to the Bayesian Dirichlet Equivalent uniform score (Heckerman et al. 1995), which guarantees that Markov equivalent DAGs are assigned the same marginal likelihood; see also Castelletti et al. (2024).

The resulting prior is conjugate to the likelihood (2.2) since, for generic dataset $ \boldsymbol{X} $, $ \boldsymbol{\theta}^{j\,|\,\mathrm{pa}(j)}_{s}\,|\,\boldsymbol{X}\sim\mathrm{Dirichlet}\big(\boldsymbol{a}^{j\,|\,\mathrm{pa}(j)}_{s}+\boldsymbol{n}_{s}^ {\mathrm{fa}(j)}\big) $ with $ \boldsymbol{n}_{s}^{\mathrm{fa}(j)}=\big(n_{(m, s)}^{\mathrm{fa}(j)},m\in\mathcal{X}_{j}\big) $. Accordingly, the posterior of $ \boldsymbol{\theta} $ is


(3.11) \begin{align*}p(\boldsymbol{\theta}\,|\,\boldsymbol{X})=\prod\limits_{j=1}^{q}\left\{\prod\limits_{s\in{\mathcal{X}}_{\mathrm{pa}(j)}}p\big(\boldsymbol{\theta}^{j\,|\,\mathrm{pa}(j)}_{s}\,|\,\boldsymbol{X}\big)\right\},\end{align*}

with each term corresponding to a Dirichlet p.d.f., so that direct sampling from $ p(\boldsymbol{\theta}\,|\,\boldsymbol{X}) $is possible. Finally, under the same prior, a marginal (i.e. integrated w.r.t. to $ \boldsymbol{\theta} $) likelihood $ m(\boldsymbol{X}\,|\,\mathcal{D})=\int p(\boldsymbol{X}\,|\,\boldsymbol{\theta},\mathcal{D})p(\boldsymbol{\theta}\,|\,\mathcal{D})\, d\boldsymbol{\theta} $ is available in closed form; see Section S1) for full details.

### 3.2. Baseline on DAGs

Let $ \mathcal{S}_{q} $ be the (discrete) space of *all* DAGs with * q * nodes. Additionally, we can restrict $ \mathcal{S}_{q} $ to a subset of DAGs satisfying some structural constraints, typically edge orientations that can be postulated in advance based on the specific real-data problem. As an instance, in our application to breast cancer data we regard age as an exogenous variable and forbid any incoming edge to it from other variables; conversely, we regard the occurrence of cardiotoxic side effect as a response variable and accordingly forbid any outgoing edge from it. Each DAG $ \mathcal{D}=(V, E) $ in $ \mathcal{S}_{q} $ can be represented through a 0 to 1 adjacency matrix $ \boldsymbol{A}^{\mathcal{D}} $, whose * (u, v) *-element $ \boldsymbol{A}^{\mathcal{D}}_{u, v}=1 $ if * (u, v)∈ E *, 0 otherwise. Additionally, let $ \boldsymbol{S}^{\mathcal{D}} $, be the adjacency matrix of the skeleton of $ \mathcal{D} $, namely the undirected graph obtained from $ \mathcal{D} $ by disregarding edges orientations. We assign for each $ u\,\gt\,v $, $ \boldsymbol{S}^{\mathcal{D}}_{u, v}\,|\,\pi\overset{\text{{iid}}}{\sim}\mathrm{Ber}(\pi) $ where * π * is a prior probability of edge inclusion. We then assume hierarchically $ \pi\sim\mathrm{Beta}(a_{\pi},b_{\pi}) $, leading to the integrated prior on DAG $ \mathcal{D} $


\begin{align*} p(\mathcal{D})\propto p\big(\boldsymbol{S}^{\mathcal{D}}\big)=\frac{\Gamma (a_{\pi}+b_{\pi})}{\Gamma(a_{\pi})\Gamma(b_{\pi})}\cdot\frac{\Gamma\big(|\boldsymbol{S}^{\mathcal{D}}|+a_{\pi}\big)\Gamma\big(q(q-1)/2-|\boldsymbol {S}^{\mathcal{D}}|+b_{\pi}\big)}{\Gamma\big(q(q-1)/2+a_{\pi}+b_{\pi}\big{)}},\end{align*}

where $ |\boldsymbol{S}^{\mathcal{D}}| $ the number of non-null elements in $ \boldsymbol{S}^{\mathcal{D}} $, corresponding to the number of edges in $ \mathcal{D} $, and * q(q-1)/2 * is the maximum number of edges in a DAG with * q * nodes. Sampling from the baseline over DAGs, as required by our MCMC sampler (Section 4), is possible through an acceptance-rejection algorithm over the space $ \mathcal{S}_{q} $; see also Section S2.

## 4. POSTERIOR INFERENCE

In this section, we detail an MCMC strategy for posterior inference of our DP mixture of categorical DAGs. This is based on a collapsed sampler with DAG parameters integrated out, and which accordingly approximates a marginal posterior over DAGs and cluster indicators $ \xi_{1},\dots , \xi_{n} $. Such output allows for inference about the clustering structure and/or the graphical structures associated with the clusters. In a second step, DAG parameters and thus causal effects can be sampled conditionally on $ \xi_{1},\dots , \xi_{n} $ based on (3.11).

### 4.1. MCMC scheme

The structure of our baseline measure (Section 3.1) is such that we can integrate out the DAG parameter $ \boldsymbol{\theta} $, which allows for the implementation of a collapsed sampler approximating the marginal posterior of $ \{\xi_{i}\}_{i\,=\,1}^{n},\alpha , \{\mathcal{D}_{k}^{\star}\}_{k\,=\,1}^{K},K $. The resulting scheme has a Gibbs-sampling structure as Algorithm 2 in Neal (2000) and implements the following steps.

#### 4.1.1. Update of cluster indicators

The full conditional of $ \xi_{i} $ can be written as


(4.12) \begin{align*}p(\xi_{i}=k\,|\,\{\boldsymbol{x}^{(l)}:l\neq i , \xi_{l}=k\},\mathcal{D}_{k}^{\star})\propto\left\{\begin{array}{@{}ll}n^{-i}_{k}\ p(\boldsymbol{x}^{(i)}\,|\,\{\boldsymbol{x}^{(l)}:l\neq i , \xi_{l}=k\},\mathcal{D}_{k}^{\star})&k=1, \dots, K\\ \alpha\ p(\boldsymbol{x}^{(i)}\,|\,\mathcal{D}_{k}^{\star})&k=K+1, \end{array}\right.\end{align*}

which corresponds to the probability that subject * i * is assigned to cluster * k *, conditionally on all the observations currently assigned to that cluster, and on $ \mathcal{D}_{k}^{\star} $. In particular, for a nonempty cluster $ k\,=\,1 , \dots, K $, the full conditional is proportional to the product between two terms: the number of observations belonging to cluster * k * (possibly excluding observation * i *), $ n^{-i}_{k}=\sum_{l\neq i}\mathds{1}\{\xi_{l}=k\} $, and the posterior predictive distribution $ p(\boldsymbol{x}^{(i)}\,|\,\{\boldsymbol{x}^{(l)}:l\neq i , \xi_{l}=k\},\mathcal {D}_{k}^{\star}) $ evaluated at $ \boldsymbol{x}^{(i)} $. For the latter, we provide in the following proposition a simple closed-form expression.

Proposition 4.1 (Posterior predictive—non-empty cluster).

For a given cluster * k *, consider the data matrix $ \boldsymbol{X}^{(k)} $ collecting the $ n_{k} $ observations $ \big\{\boldsymbol{x}^{(l)}:\xi_{l}=k\big\} $, and an observation $ \boldsymbol{x}^{(i)} $. Then, the posterior predictive of $ \boldsymbol{x}^{(i)} $ given $ \{\boldsymbol{x}^{(l)}:l\neq i , \xi_{l}=k\} $ is

(4.13) \begin{align*}p(\boldsymbol{x}^{(i)}\,|\,\{\boldsymbol{x}^{(l)}:l\neq i , \xi_{l}=k\},\mathcal{D}_{k}^{\star})=\prod_{j=1}^{q}\left\{\frac{a/|{\mathcal{X}}_{\mathrm{fa}(j)}|+{}_{k}n^{\mathrm{fa}(j)}_{(\tilde{m}_{j},\tilde{s}_{j})} -\mathds{1}\{\xi_{i}=k\}}{a/|{\mathcal{X}}_{\mathrm{pa}(j)}|+{}_{k}n^{\mathrm{pa}(j)}_{\tilde{s}_{j}}-\mathds{1}\{\xi_{i}=k\}}\right\}\end{align*}

where $ \tilde{m}_{j}=\boldsymbol{x}^{(i)}_{j},\tilde{s}_{j}=\boldsymbol{x}^{(i)}_{\mathrm{pa}(j)} $ and

\begin{align*} {}_{k}n^{\mathrm{fa}(j)}_{(\tilde{m}_{j},\tilde{s}_{j})}=\sum\limits_{l : \xi_{l}=k}\mathds{1}\big\{\boldsymbol{x}^{(l)}_{\mathrm{fa}(j)}=(\tilde{m}_{j},\tilde{s}_{j})\big\},\quad{}_{k}n^{\mathrm{pa}(j)}_{\tilde{s}_{j}}=\sum\limits_{l : \xi_{l}= k}\mathds{1}\big\{\boldsymbol{x}^{(l)}_{\mathrm{pa}(j)}=\tilde{s}_{j}\big{\}}.\end{align*}

Proof. See Section S3. ▪

The second expression of (4.12) considers the case of a (new) empty cluster $ k\,=\,K\,+\,1 $, where the DAG $ \mathcal{D}_{K\,+\,1} $ is sampled from the baseline over $ \mathcal{S}_{q} $. In such a case, the full conditional is proportional to the product of the concentration parameter * α * and a posterior predictive which reduces to the marginal likelihood (prior predictive) of a cluster containing subject * i * only. A related closed-form expression is provided by the following proposition.

Proposition 4.2 (Posterior predictive—empty cluster). For a new cluster $ k\,=\,K\,+\,1 $, the posterior predictive of $ \boldsymbol{x}^{(i)} $ coincides with the marginal likelihood and is given by

(4.14) \begin{align*} p(\boldsymbol{x}^{(i)}\,|\,\mathcal{D}_{k}^{\star})=\prod_{j=1}^{q}\frac{1}{|\mathcal{X}_{j}|}.\end{align*}

Proof. See Section S3. ▪

#### 4.1.2. *Update of* $ \boldsymbol{\alpha} $

Under the DP prior, the full conditional distribution of * α * reduces to $ p(\alpha\,|\, K)\propto p(K\,|\,\alpha)p(\alpha) $, where in particular


\begin{align*} p(K\,|\,\alpha)\propto c_{n}(K)\alpha^{K}\frac{\Gamma(\alpha)}{\Gamma(\alpha+n)}\end{align*}

is the prior on the number of clusters induced by the DP and $ c_{n}(K) $ is a normalizing constant not involving * α *. Sampling from $ p(\alpha\,|\, K) $ can be done by augmenting the distribution through an auxiliary variable $ \eta\sim\mathrm{Beta}(\alpha\,+\,1, n) $. It can be shown (Escobar and West 1995) that under the prior $ \alpha\sim\mathrm{Gamma}(c, d) $ the full conditional of $ \alpha\,|\, K , \eta $ corresponds to a mixture of Gamma distributions, specifically


\begin{align*}\alpha\,|\,\eta, K\sim g\cdot\mathrm{Gamma}(c+K, d-\log\eta)+(1-g)\cdot\mathrm{Gamma}(c+K-1, d-\log\eta),\end{align*}

where $ g/(1-g)=(c\,+\,K-1)/n(d-\log\eta) $.

#### 4.1.3. Update of DAGs and sampling of DAG parameters

Let * K * be the number of clusters and $ \xi_{1},\dots , \xi_{n} $ the cluster indicators, with each $ \xi_{i}\in\{1 , \dots, K\} $. For a given $ k\in\{1 , \dots, K\} $, let $ \{\boldsymbol{x}^{(i)}:\xi_i=k\} $ be the set of observations currently assigned to cluster * k *, and $ \boldsymbol{X}^{(k)} $ the implied $ (n_{k},q) $ data matrix; see also (3.8). Without loss of generality, consider a generic cluster and omit for simplicity subscripts * k * from $ \mathcal{D}_{k}^{\star} $ and $ \boldsymbol{X}^{(k)} $. Update of DAG $ \mathcal{D} $ is performed through a Metropolis Hastings step where a DAG $ \widetilde{\mathcal{D}} $ is sampled from a proposal distribution $ q(\widetilde{\mathcal{D}}\,|\,\mathcal{D}) $ conditionally on a current DAG $ \mathcal{D} $ and it is accepted with probability $ \alpha_{\widetilde{\mathcal{D}}}=\min\{1; r_{\widetilde{\mathcal{D}}}\} $ with


(4.15) \begin{align*} r_{\widetilde{\mathcal{D}}}=\frac{m(\boldsymbol{X}\,|\,\widetilde{\mathcal{D}})}{m(\boldsymbol{X}\,|\,\mathcal{D})}\cdot\frac{p(\widetilde{\mathcal{D}})}{p(\mathcal{D})}\cdot\frac{q(\mathcal{D}\,|\,\widetilde{\mathcal{D}})}{q(\widetilde{\mathcal{D}}\,|\,\mathcal{D})};\end{align*}

see Section S2 for full details.

Finally, conditionally on DAGs $ \mathcal{D}^\star_{1},\dots , \mathcal{D}^\star_{K} $ and indicators $ \xi_{1},\dots , \xi_{n} $, we can sample each DAG parameter $ \boldsymbol{\theta}^\star_{k} $ based on (3.11), corresponding to the posterior of $ \boldsymbol{\theta}^\star_{k} $ which is available in closed-form as a product of Dirichlet probability functions; see also Section 3.1.

### 4.2. Posterior summaries

Output of the MCMC scheme is a collection of cluster indicators, DAGs, and DAG parameters approximately drawn from the posterior distribution of our DP mixture model. Starting from this output, we can provide posterior summaries regarding clustering, DAG structures, as well as DAG-model parameters. Specifically, let $ K^{(s)} $ be the number of clusters at MCMC iteration * s *, and $ \xi_{i}^{(s)} $, $ i\,=\,1 , \dots, n $, $ \mathcal{D}_{k}^{(s)} $, $ \boldsymbol{\theta}_{k}^{(s)} $, $ k\,=\,1 , \dots, K^{(s)} $, the corresponding realizations of the three sets of parameters. For clustering purposes, we first recover an * (n, n) * posterior similarity matrix $ \boldsymbol{S} $, with $ (i, i^{\prime}) $-element $ \boldsymbol{S}_{i, i^{\prime}} $ corresponding to the (estimated) posterior probability that individuals * i * and $ i^{\prime} $ are assigned to the same cluster, namely


(4.16) \begin{align*}\widehat{p}(\xi_{i}=\xi_{i^{\prime}}\,|\,\boldsymbol{X})=\frac{1}{S}\sum_{s=1} ^{S}\mathds{1}\left\{\xi_{i}^{(s)}=\xi_{i^{\prime}}^{(s)}\right\}.\end{align*}

A point estimate of the clustering structure, $ \widehat{\boldsymbol{c}} $, can be recovered by assigning individuals * i * and $ i^{\prime} $ to the same cluster if $ \widehat{p}(\xi_{i}=\xi_{i^{\prime}}\,|\,\boldsymbol{X}) $ exceeds a given threshold, say $ z\,=\,0.5 $. As an alternative, a clustering estimate can be obtained as the partition minimizing an expected loss function, such as the Variation of Information (VI); see in particular Wade and Ghahramani (2018).

From the same MCMC output, we can recover for each subject * i * a * (q, q) * matrix collecting estimates of the Posterior Probabilities of edge Inclusion (PPIs). Let $ K^{(s)} $ be the number of allocated clusters at MCMC iteration * s *, $ \mathcal{D}_{1}^{(s)},\dots , \mathcal{D}_{K^{(s)}}^{(s)} $ the corresponding DAGs, and recall that each $ \xi_{i}^{(s)}\in\{1 , \dots, K^{(s)}\} $. For a given subject * i * and edge * u→ v *, its PPI is estimated as


(4.17) \begin{align*}\widehat{p}_{i}(u\rightarrow v\,|\,\boldsymbol{X})=\frac{1}{S}\sum_{s=1}^{S}\mathds{1}\left\{u\rightarrow v\in\mathcal{D}^{(s)}_{\xi_{i}^{(s)}}\right\},\end{align*}

which corresponds to the proportion of DAGs assigned to subject * i * in the chain that contain the directed edge * u→ v *. Finally, a graph estimate at subject-specific level, say $ \widehat{\mathcal{D}}_{i} $, can be obtained by including those edges for which $ \widehat{p}_{i}(u\rightarrow v\,|\,\boldsymbol{X}) > z $ for * z∈(0,1) *, eg $ z\,=\,0.5 $.

Recall now the definition of causal effect $ \gamma_{y, h}(\boldsymbol{\theta}) $ provided in (2.6) and assume that the intervened variable and response, $ X_{h} $ and * Y * respectively, are given so that we can omit them from the notation. The MCMC sample $ \Big\{\gamma_{i}^{(s)}=\gamma\Big(\boldsymbol{\theta}_{\xi_{i}^{(s)}}^{(s)}\Big),s\,=\,1 , \dots, S\Big\} $ provides an approximate posterior of the subject-specific causal effect parameter $ \gamma_{i}=\gamma(\boldsymbol{\theta}_{i}) $, for any $ i\,=\,1 , \dots, n $. In addition, a Bayesian Model Averaging (BMA) estimate of $ \gamma_{i} $ is given by


(4.18) \begin{align*}\widehat{\gamma_{i}}=\frac{1}{S}\sum_{s=1}^{S}\gamma_{i}^{(s)}.\end{align*}

The resulting collection $ \{\widehat{\gamma_{1}},\dots , \widehat{\gamma}_{n}\} $ provides estimates of causal effects at individual level, which also naturally account for DAG-model uncertainty through BMA. Also notice that subject-specific quantities, including $ \gamma_{i} $ and its corresponding estimate $ \widehat{\gamma}_{i} $, are not subject to label switching issues because they do not depend anymore on the specific label indexing the cluster-membership of subject * i *.

## 5. ANALYSIS OF BREAST CANCER DATA

### 5.1. Dataset and model implementation

In this section, we analyze a dataset of $ n\,=\,404 $ women diagnosed with HER2+ breast cancer and treated with potentially cardiotoxic therapies based on monoclonal antibodies (trastuzumab) and chemotherapy drugs (anthracyclines). Variables in the dataset include demographic and physical features (eg age, BMI); risk factors, such as diagnosis of hypertension, dyslipidemia; past cardiac diseases, including cardiac insufficiency, ischemic cardiomyopathy, arrhythmia, and valvulopathy. In addition, the dataset provides information regarding treatments, antiHER2 monoclonal therapy (antiHER2) and/or anthracyclines (AC). Since these treatment indicators are not mutually exclusive and patients may receive both or none of the two, we include them as two distinct variables in the model.

Finally, the target response variable corresponds to the Cancer Therapy-Related Cardiac Dysfunction (CTRCD), a binary outcome indicating the occurrence (1) or not (0) of cardiac dysfunction. The original dataset is provided as a supplement to Piñeiro-Lamas et al. (2023) and available as Supplementary Material to our paper. A summary of all categorical variables included in our analysis is also reported in Table 1. Notably, all the original variables are categorical by nature, with the exception of BMI, age, heart rate, and Left Ventricular Ejection Fraction (LVEF) (ie the fraction of blood pumped out of the left ventricle with each heartbeat). Each of them has been discretized into a categorical variable with levels $ \{0,1,2\} $ corresponding to low, mid, and high values of the original variable. Descriptive summaries of the data are reported in Section S6.

**Table 1:** Breast cancer data. ID, description, and levels of each categorical variable included in the analysis.

**ID**	**Description**	**Levels**
CTRCD	Cancer therapy-related cardiac dysfunction	0 (no), 1 (yes)
AC	Drug therapy based on anthracycline	0 (no), 1 (yes)
antiHER2	Therapy based on monoclonal antibodies	0 (no), 1 (yes)
HTA	Hypertension	0 (no), 1 (yes)
DL	Dyslipidemia	0 (no), 1 (yes)
DM	Diabetes mellitus	0 (no), 1 (yes)
smoker	Smoker	0 (no), 1 (yes)
exsmoker	Ex smoker	0 (no), 1 (yes)
RTprev	Previous thorax radiotherapy	0 (no), 1 (yes)
CIprev	Previous cardiac insufficiency	0 (no), 1 (yes)
ICMprev	Previous ischemic cardiomyopathy	0 (no), 1 (yes)
ARRprev	Previous arrhythmia	0 (no), 1 (yes)
cxvalv	Valve surgery	0 (no), 1 (yes)
VALVprev	Previous valvulopathy	0 (no), 1 (yes)
BMI	Body mass index (kg/m^2^)	0 (BMI * ≤ 25 *),
		1 (* 25 < * BMI * ≤ 35 *),
		2 (BMI * > 35 *)
age	Age (yrs)	0 (age * ≤ 45 *),
		1 (* 45 < * age * ≤ 65 *),
		2 (age * > 65 *)
heart rate	Heart rate (beats per minute)	0 (HR * ≤ 60 *),
		1 (* 60 < * HR * ≤ 100 *),
		2 (HR * > 100 *)
heart rhythm	Atrial fibrillation	0 (no), 1 (yes)
LVEF	Left ventricular ejection fraction (%)	0 (LVEF * ≤ 50 *),
		1 (* 50 < * LVEF * ≤ 70 *),
		2 (LVEF * > 70 *)

While the cardiotoxic effects of the available oncological therapies have been established in the literature, the occurrence of CTRCD can still vary substantially among patients because of both observed features (such as risk factors) or even unobserved characteristics. Accordingly, it is of interest to quantify causal effects w.r.t. the occurrence of CTRCD at individual-level, which is crucial for the development and administration of appropriate antiHER2 therapies.

Given the structure of the dataset, we constrain the adjacency matrix of DAGs in such a way that CTRCD can only (potentially) be a response, ie no outgoing edges are allowed, and treat age, BMI, smoker, ex smoker as exogenous variables, ie with no incoming edges from other nodes, while possible links/dependencies between them are allowed. Moreover, we assume that the absence/presence of risk factors can determine the administration of a therapy (AC, antiHER2), while the converse is not possible. We implement our mixture model by running the MCMC scheme for $ S\,=\,100\,000 $ iterations, which include a burn-in period of * 10000 * draws that are discarded from the posterior analysis. With regard to hyperparameters, we fix $ c\,=\,3, d\,=\,1 $ in the Gamma prior on the DP concentration parameter * α *. This choice centers the prior around a moderate * K *-value, while allowing substantial variability, and thus provides a weakly informative choice that does not strongly constrain the number of clusters but avoids extreme values that may lead to overly sparse or diffuse partitions. The common hyperparameter * a * on the collection of Dirichlet priors on $ \boldsymbol{\theta}^{j\,|\,\mathrm{pa}(j)}_{s} $ is instead fixed as $ a\,=\,1 $, a standard choice since the resulting marginal likelihood coincides with the BDeu score commonly used in alternative (frequentist) structure learning approaches, such as the Hill Climbing algorithm (see also Section S5). In the hierarchical prior on DAGs we fix $ a_{\pi}=1 $, $ b_{\pi}=2q $, with $ q\,=\,19 $ the number of variables, reflecting an a priori assumption of sparsity in the graph space. To assess the convergence of our algorithm we also run two independent MCMC chains; results suggest an overall agreement in terms of clustering (evaluated through posterior similarity matrices), structure learning (based on estimated PPIs) and posterior distribution of causal-effect parameters. Results relative to all such quantities are presented and discussed in the following sections.

### 5.2. Clustering

**Figure 1:**
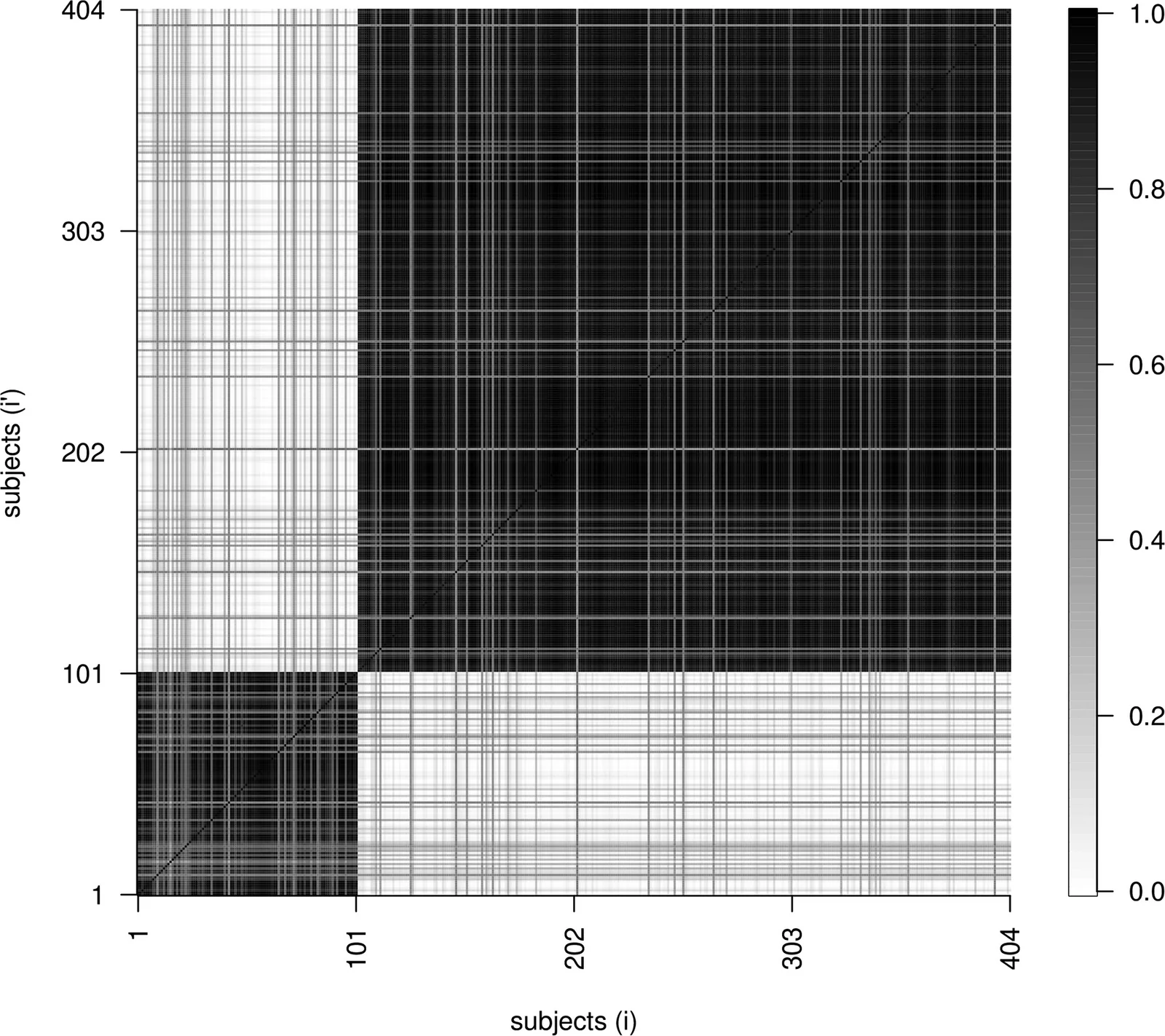
Breast cancer data. Estimated posterior similarity matrix, with individuals arranged according to the clustering structure estimated via the posterior expectation of the VI (clusters labeled as $ \widehat{\boldsymbol{c}}_{1} $ and $ \widehat{\boldsymbol{c}}_{2} $, respectively).

**Figure 2:**
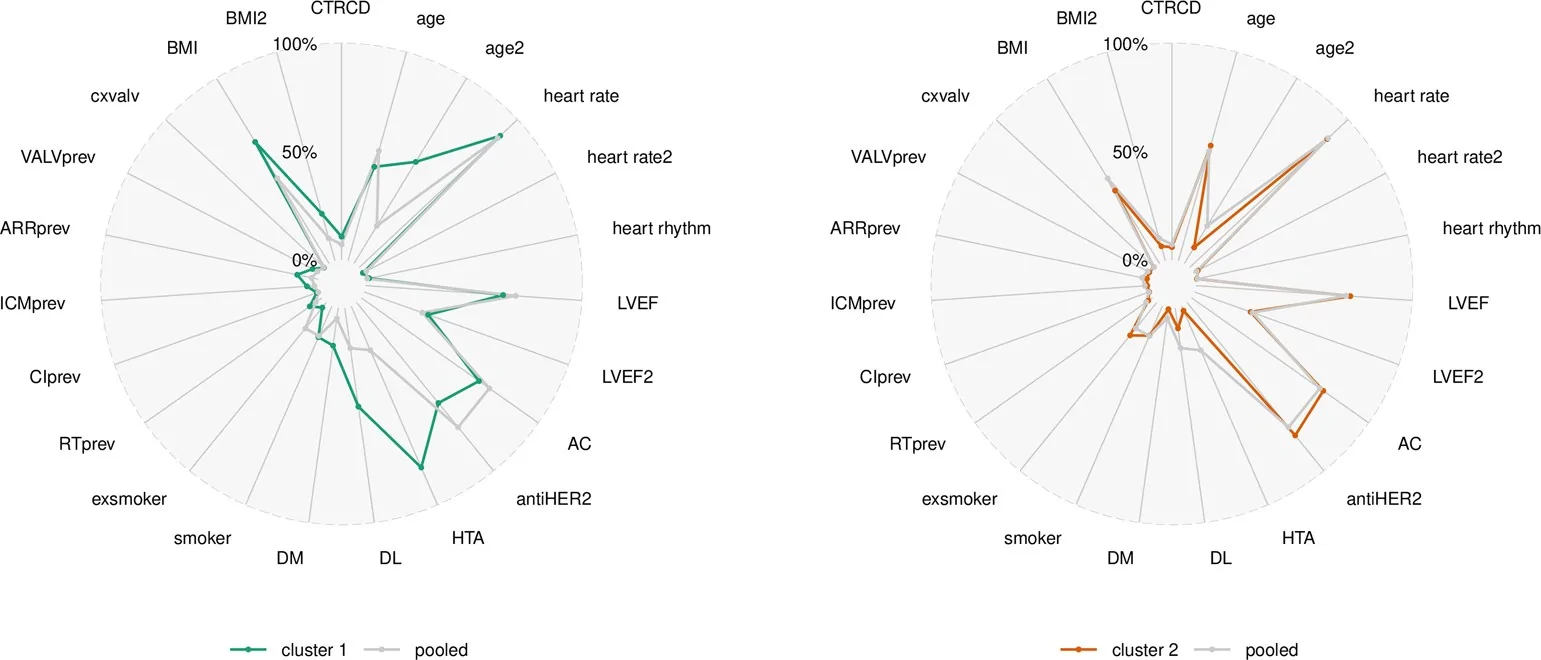
Breast cancer data. Spider plots for the comparison of the empirical marginal distribution of each variable between estimated cluster (colored line) and pooled dataset (gray line). Left and right panels correspond to clusters 1 and 2, respectively.

We summarize the clustering structure learned by our model by building an * (n, n) * posterior similarity matrix; see (4.16). From the latter, we recover a point estimate of the clustering based on the minimum posterior expectation of the VI (Wade and Ghahramani 2018); see also Section 4.2. As the result, we obtain two clusters, that we label as $ \widehat{\boldsymbol{c}}_{1} $ and $ \widehat{\boldsymbol{c}}_{2} $, whose sizes are $ n_{1}=101 $ and $ n_{2}=303 $, respectively. The posterior similarity matrix is represented as a heatmap in Fig. 1, with individuals arranged according to the estimated clusters, specifically those assigned to $ \widehat{\boldsymbol{c}}_{1} $ first and then those in $ \widehat{\boldsymbol{c}}_{2} $. The two-cluster structure is pretty evident from the matrix since the probabilities of membership to the same group approach value 1 (0) for individuals assigned to the same (to a different) estimated cluster. We then investigate differences between the estimated clusters by comparing the empirical (marginal) distribution of each variable across $ \widehat{\boldsymbol{c}}_{1} $ and $ \widehat{\boldsymbol{c}}_{2} $. For each cluster, we provide a graphical representation based on a spider plot, which includes for each categorical binary variable the proportion (percentage values) of observations corresponding to level labeled as 1 of the variable. For categorical variables with three levels (e.g. age, see also Table 1) we instead include in the plot both the proportion of 1 (age) and of 2 (age2) values. All such proportions are reported as colored points joined by lines in each graph; see Fig. 2. Additionally, each plot includes the same proportions as obtained from the pooled sample, namely when no clustering is considered, that are instead represented as grey dots joined by grey lines. While for cluster 2 (right-side plot) the cluster-proportions are almost aligned with those computed on the pooled dataset, cluster 1 presents a few peculiarities. In particular, patients included in cluster 1 are in general older, as it appears from the higher frequency associated with variable age2, and characterized by a higher BMI index. Additionally, the proportion of patients suffering from hypertension (HTA), dyslipidemia (DL), and diabetes mellitus (DM) is in general higher in comparison with the pooled dataset, than those in cluster 2. Importantly however, differences may emerge from the *joint* distribution, and specifically in the dependence (DAG) structure for patients assigned to different estimated clusters. To this end, in the next section we provide results relative to structure learning, which is carried out at subject-specific level.

### 5.3. Structure learning

We provide an overall summary of our findings in terms of heterogeneous dependence structures by considering the Structural Hamming Distance (SHD) between subject-specific DAGs. The latter corresponds to the number of graph modifications (edge insertions, deletions or flips) needed to transform one graph into another. Specifically, we build an * (n, n) * matrix $ \widehat{\boldsymbol{D}} $, with the * n * individuals ordered according to the same point-estimate of the clustering partition (Section 5.2), whose $ (i, i^{\prime}) $-element is an MCMC-based estimate of the SHD between subject-specific DAGs $ \widehat{\mathcal{D}}_{i} $ and $ \widehat{\mathcal{D}}_{i^{\prime}} $, namely


\begin{align*}\widehat{\boldsymbol{D}}_{i, i^{\prime}}=\frac{1}{S}\sum_{s=1}^{S}\mathrm{SHD}\left(\mathcal{D}^{(s)}_{\xi_{i}^{(s)}},\mathcal{D}^{(s)}_{\xi_{i\prime}^{(s)}}\right).\end{align*}

**Figure 3:**
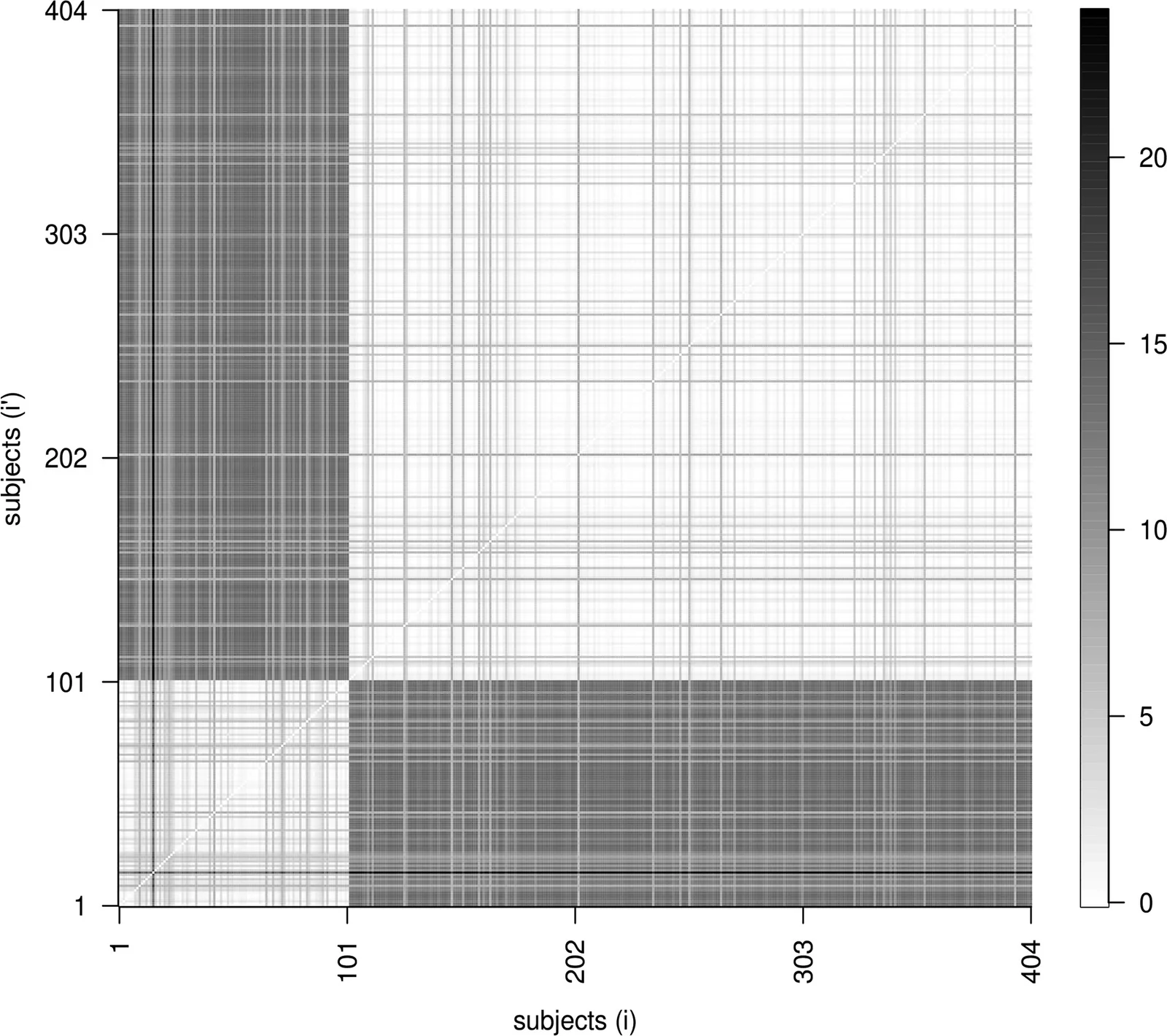
Breast cancer data. Heatmap of the estimated SHD between each pair of subject-specific DAGs.

**Figure 4:**
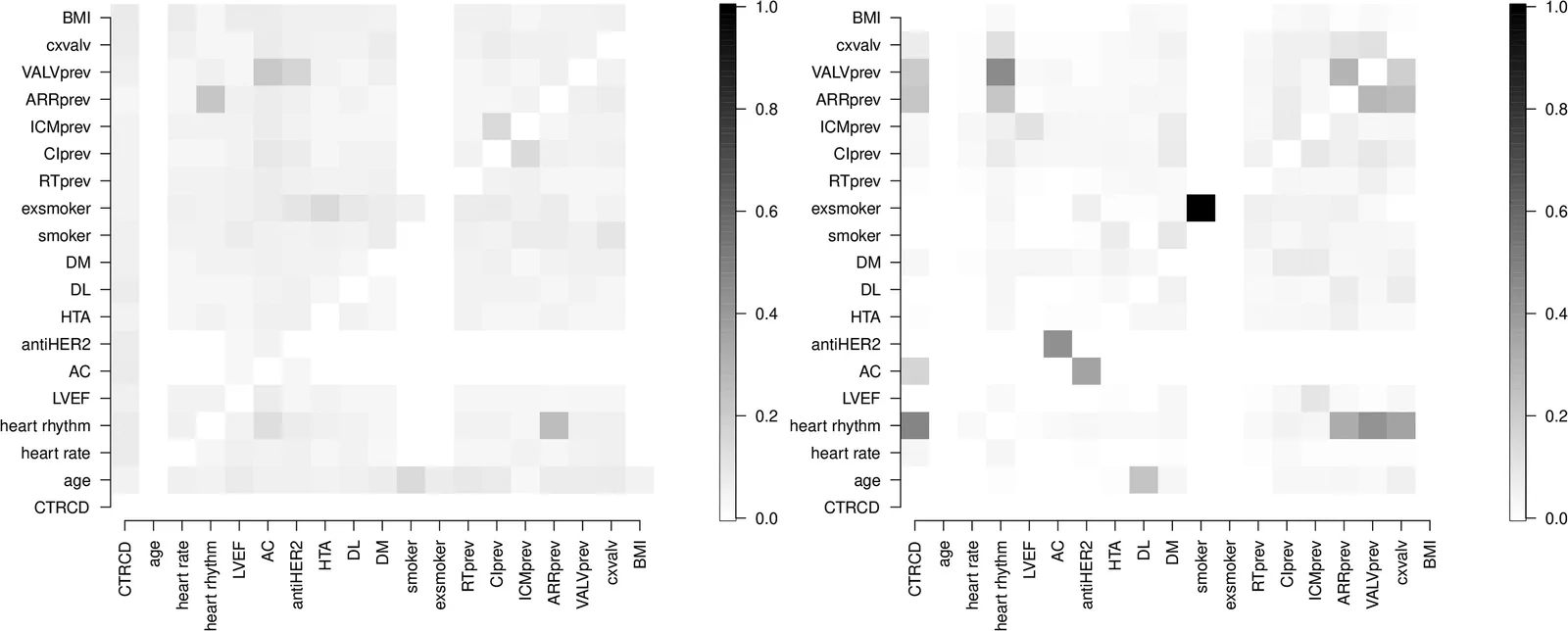
Breast cancer data. Heatmaps of posterior probabilities of edge inclusion for two subject-specific graphs. Left map corresponds to 1 subject in estimated cluster 1; right map to 1 subject in cluster 2.

Accordingly, lower values of the index correspond to stronger similarities between the two individuals in terms of the underlying dependence structure. The resulting matrix is represented as a heatmap in Fig. 3. A direct comparison with the posterior similarity matrix in Fig. 1 shows that individuals having higher probabilities of sharing the same cluster-membership are also characterized by DAG structures that are closer in terms of SHD. The opposite behavior is observed for those individuals assigned to distinct estimated clusters. Furthermore, according to (4.17), we provide for each subject $ i\,=\,1 , \dots, n $ an estimate of the Posterior Probabilities of edge Inclusion (PPIs), that we collect in a * (q, q) * matrix. For two randomly chosen subjects, whose memberships are estimated to be cluster 1 and cluster 2, respectively, the corresponding PPIs are reported as heatmaps in Fig. 4. The underlying dependence structures are both characterized by sparsity. This is much more evident in subject from cluster 1, where PPIs are more uniform and there are no edges whose PPI exceeds the 0.5 threshold. Differently, the heatmap from cluster 2 shows a few variables that are more strongly related to the outcome CTRCD, specifically AC, together with a few risk factors, in particular hearth rhythm, VALVprev, ARRprev. Accordingly, we expect such differences to imply heterogeneous causal effects across subjects; see the next section.

### 5.4. Causal effects

**Figure 5:**
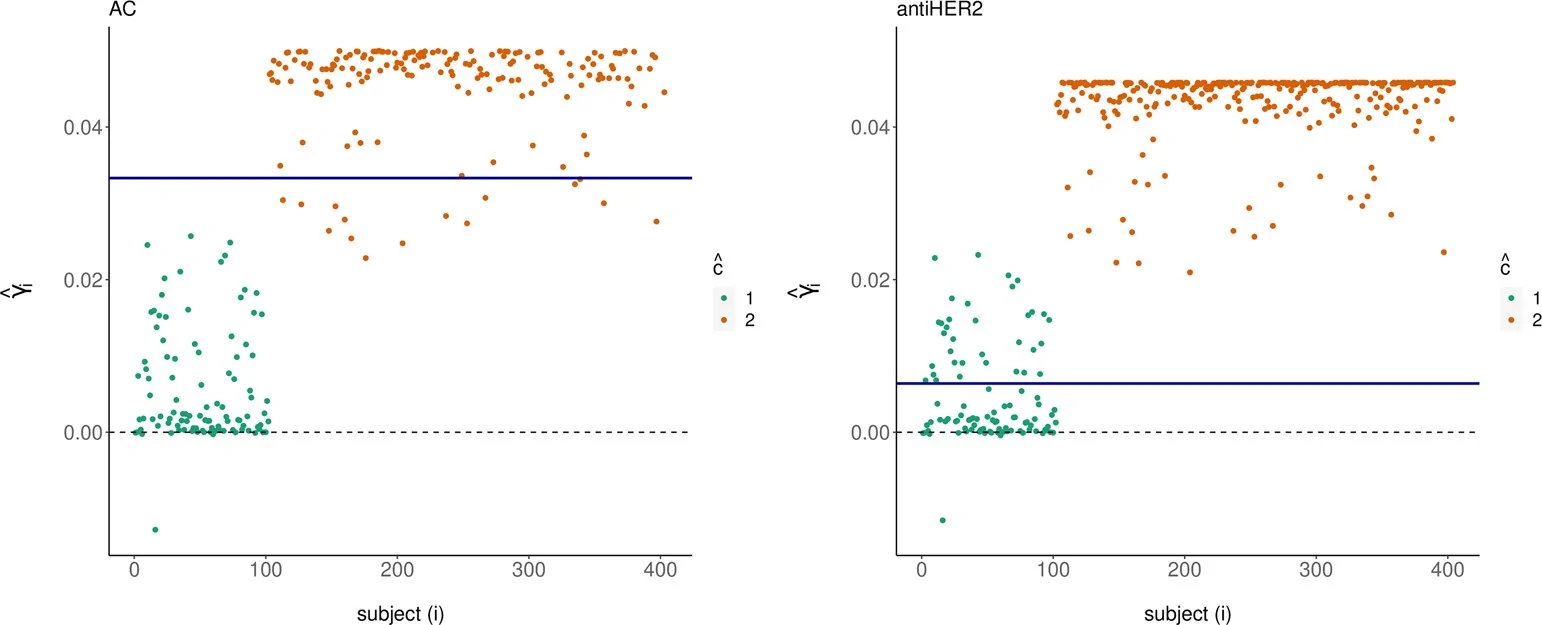
Breast cancer data. BMA estimates of subject-specific causal effects, with individuals arranged according to the estimated clustering structure. Cluster $ \widehat{\boldsymbol{c}}_{1} $ is shown in green and cluster $ \widehat{\boldsymbol{c}}_{2} $ in orange. Blue lines correspond to causal-effect estimates obtained under a one-group naive strategy disregarding heterogeneity.

The ultimate goal of our analysis is to quantify the causal effect of anticancer therapies on the occurrence of cardiotoxicity. Patients in the study were treated with therapies based on anthracycline (AC) or trastuzumab (antiHER2); additionally, a few of them did not receive any of the treatments, while other individuals received both. We consider in what follows the (marginal) causal effect on the occurrence of cardiotoxicity (variable CTRCD) implied by the administration of either AC or antiHER2. Although not addressed in this work, estimation of causal effects resulting from a *joint* administration of antiHER2 and AC would be possible, particularly by adapting to categorical data the framework of Nandy et al. (2017), which is tailored to Gaussian DAGs; see also Castelletti and Mascaro (2021). For each therapy, we recover from our MCMC output the posterior distribution of the * n * subject-specific causal-effect parameters. Each is then summarized through BMA estimates according to (4.18). Our results are summarized in the two scatterplots of Fig. 5, each reporting BMA causal-effect estimates (*y*-axis) computed across individuals (*x*-axis) and with subjects arranged according to the estimated clustering with two groups (Section 5.2). One can appreciate the heterogeneity in the estimates, with individuals assigned to the same cluster sharing similar values, except for a few patients in each group. Interestingly, these subjects are also characterized by a higher uncertainty in cluster allocation between either group 1 or 2; see in particular the posterior similarity matrix in Fig. 1. As an interesting result, AC and antiHER2 treatments in general increase the probability of CTRCD occurrence for individuals assigned to cluster 2, while the effect is less pronounced, or even null, for cluster 1. Notably, cluster 1 is characterized by older patients and with a higher prevalence of some risk factors; see in particular Fig. 2. Accordingly, in such patients, the occurrence of cardiotoxicity might be due to the presence of such risk factors, that may cause cardiac diseases, rather than implied by the therapy itself. Therefore, the *direct* effect of AC and antiHER2 therapies is lower in comparison with the same estimates in cluster 2.

Finally, to emphasize the role played by population heterogeneity in causal effect estimation, we compare our results with those based on an alternative one-group naive strategy, corresponding to a standard categorical DAG model in which all individuals are assigned to the same cluster. In this case, causal effect estimates are uniform across subjects and are included as horizontal lines in the two plots. Each resulting estimate is approximately an average of cluster-specific causal estimates, suggesting that a causal effect analysis that disregards population heterogeneity would over- or under- estimate the risk of CTRCD development across individuals.

## 6. CONCLUSION

We proposed a modeling framework for personalized causal inference that applies to multivariate categorical data. Our methodology is based on a DP mixture of categorical DAGs which allows to cluster subjects characterized by similar patterns of dependencies into homogeneous groups, and to provide estimates of causal effects at personalized, namely subject-specific, level. When adopted for clustering purposes, our method outperforms benchmark strategies that do not account for dependence relations in the joint distribution of variable; see in particular our results in Section S4. Most importantly, our causal-effect analysis shows that approaches neglecting possible population heterogeneity can provide misleading estimates of causal effects. With regard to the analysis of breast cancer data, the probability of cardiotoxic side effects implied by anticancer therapies could be underestimated for several patients, with serious consequences in clinical decision-making for optimal therapy administration.

Limitations of the present work primarily relate to scalability. As the number of nodes increases, achieving convergence of the MCMC algorithm becomes more challenging, due to the necessity of exploring the space of DAGs, which grows super-exponentially with the number of nodes. In addition, our model assumes that all variables are categorical. While this assumption is appropriate for many biomedical applications, such as those involving treatment indicators, disease diagnoses, and risk factors, real-world datasets may contain variables of different types. A natural direction for future research is to extend the model to handle mixed data, thus accommodating both categorical and continuous variables within a unified framework. Specifically in a biomedical setting, one can collect besides categorical clinical features the expression levels of genes that are involved in the progression of the disease. Causal inference methods that integrate such biological information can provide a more precise quantification of causal effects for the development of personalized therapies. More in general, multivariate models that can manage mixed data would be valuable for clustering purposes too, as several real-world applications frequently involve data of various types. Mixed-data represent an interesting framework for graphical modeling which has been addressed in a few works, although without accounting for population heterogeneity; see for instance Castelletti (2024). From an applied perspective, the breast cancer dataset provided by Piñeiro-Lamas et al. (2023) includes Tissue Doppler Imaging (TDI) data, which measure the rate of contraction and relaxation of the cardiac muscle. TDI measurements can be treated as functional data, which whenever included in our analysis could help identifying sub-groups of patients who experienced different side effects of anti-HER2 therapies depending on the presence of cardiac dysfunctions. The inclusion of functional variables in our framework presents challenges that are related to the development of a DAG-based model. As a starting point, Qiao et al. (2019) propose a frequentist method to learn dependencies across functional variables, which is based on undirected graphical models and lasso-type penalization techniques.

## Supplementary Material

kxag026_Supplementary_Data

## Data Availability

The dataset analyzed in Section 5 is publicly available at https://github.com/LauraFerrini/DP-Mixture-categorical-DAGs.

## Supplementary material

Supplementary material is available at *Biostatistics Journal* online. Code implementing our methodology is publicly available at https://github.com/LauraFerrini/DP-Mixture-categorical-DAGs.
