# Supplementary material for: Bayesian nonparametric mixtures of categorical directed graphs for personalized causal inference
Source: Biostatistics. 2026 Jul 28;27(1):kxag026. doi: 10.1093/biostatistics/kxag026 (PMC13407445; doi:10.1093/biostatistics/kxag026)
Supplement: kxag026_Supplementary_Data [file kxag026_supplementary_data.pdf]

Supplementary Material for
Bayesian nonparametric mixtures of categorical directed graphs for personalized causal inference

Federico Castelletti and Laura Ferrini

This Supplementary Material is organized into various sections. Section 1 provides details about the marginal likelihood of a categorical DAG model. Section 2 summarizes the construction of the proposal distribution over the DAG space, as required by our MCMC sampler. Section 3 contains the proofs of Propositions 1 and 2 for the full conditional distributions of cluster indicators. Sections 4 and 5 present simulation studies and comparisons of our methodology with alternative approaches for clustering, structure learning and heterogeneous causal inference. Finally, Section 6 presents descriptive summaries and MCMC diagnostics for our application to breast cancer data, and discusses the computational time of our MCMC scheme.

1. Details on the DAG marginal likelihood

Consider a DAG $\mathcal{D}$ and an (n,q) data matrix $\boldsymbol{X}$ collecting n q-variate categorical observations $\boldsymbol{x}^{(i)} = (x_1^{(i)},\dots,x_q^{(i)})^\top, i=1,\dots,n$. Assume the likelihood function $p(\boldsymbol{X}\,|\,\boldsymbol{\theta},\mathcal{D})$ and prior $p(\boldsymbol{\theta}\,|\,\mathcal{D})$ as in Section 1 and 3.1 of the main paper respectively. Then, the marginal likelihood of a categorical DAG model is given by

$$m(\boldsymbol{X}\,|\,\mathcal{D}) = \int p(\boldsymbol{X}\,|\,\boldsymbol{\theta},\mathcal{D})\; p(\boldsymbol{\theta}\,|\,\mathcal{D})\; d\boldsymbol{\theta} = \prod_{j=1}^{q}\left\{\prod_{s\in\mathcal{X}_{\mathrm{pa}(j)}} \frac{h\big(\boldsymbol{a}_s^{j\,|\,\mathrm{pa}(j)}\big)}{h\big(\boldsymbol{a}_s^{j\,|\,\mathrm{pa}(j)}+\boldsymbol{n}_s^{\mathrm{fa}(j)}\big)}\right\}, \tag{1}$$

where in particular

$$h\big(\boldsymbol{a}_s^{j\,|\,\mathrm{pa}(j)}\big) = \frac{\Gamma\Big(\sum_{m\in\mathcal{X}_j} a_{m\,|\,s}^{j\,|\,\mathrm{pa}(j)}\Big)}{\prod_{m\in\mathcal{X}_j}\Gamma\Big(a_{m\,|\,s}^{j\,|\,\mathrm{pa}(j)}\Big)},$$

$$h\big(\boldsymbol{a}_s^{j\,|\,\mathrm{pa}(j)}+\boldsymbol{n}_s^{\mathrm{fa}(j)}\big) = \frac{\Gamma\Big(\sum_{m\in\mathcal{X}_j} a_{m\,|\,s}^{j\,|\,\mathrm{pa}(j)} + n_{(m,s)}^{\mathrm{fa}(j)}\Big)}{\prod_{m\in\mathcal{X}_j}\Gamma\Big(a_{m\,|\,s}^{j\,|\,\mathrm{pa}(j)} + n_{(m,s)}^{\mathrm{fa}(j)}\Big)}$$

are the prior and posterior normalizing constants respectively. See Castelletti et al. (2024) for details. Moreover, under the default choice $a_{m\,|\,s}^{j\,|\,\mathrm{pa}(j)} = a/|\mathcal{X}_j|$, Equation (1) reduces to

$$m(\boldsymbol{X}\,|\,\mathcal{D}) = \prod_{j=1}^{q}\left\{\prod_{s\in\mathcal{X}_{\mathrm{pa}(j)}} \frac{\Gamma\big(a/|\mathcal{X}_{\mathrm{pa}(j)}|\,\big)}{\Gamma\Big(a/|\mathcal{X}_{\mathrm{pa}(j)}| + n_s^{\mathrm{pa}(j)}\Big)} \prod_{m\in\mathcal{X}_j} \frac{\Gamma\Big(a/|\mathcal{X}_{\mathrm{fa}(j)}| + n_{(m,s)}^{\mathrm{fa}(j)}\Big)}{\Gamma\big(a/|\mathcal{X}_{\mathrm{fa}(j)}|\big)}\right\}. \tag{2}$$

2. Proposal distribution and sampling from the baseline over DAGs

We provide details about the construction of a proposal distribution to explore the space of DAGs. This is required by our MCMC sampler to update DAGs through a Metropolis Hastings step and to

sample from the baseline over DAGs in the update of cluster indicators; see in particular Section 4.1 of our paper.

Consider $\mathcal{D} \in \mathcal{S}_q$ where $\mathcal{S}_q$ is a collection of DAGs, such as the space of *all* DAGs having q nodes. To update $\mathcal{D}$, we first draw a new DAG $\widetilde{\mathcal{D}}$ from a proposal distribution which is based on three types of operators that locally modify $\mathcal{D}$: insert a directed edge (InsertD $u \to v$ for short), delete a directed edge (DeleteD $u \to v$) and reverse a directed edge (ReverseD $u \to v$). We then construct the set of valid operators $\mathcal{O}_{\mathcal{D}}$, that is operators whose resulting graph is in $\mathcal{S}_q$. Finally, we propose $\widetilde{\mathcal{D}}$ by uniformly sampling an element in $\mathcal{O}_{\mathcal{D}}$ and applying it to $\mathcal{D}$. Since there is a one-to-one correspondence between each operator and the resulting DAG, the probability of transition is $q(\widetilde{\mathcal{D}} \,|\, \mathcal{D}) = 1/|\mathcal{O}_{\mathcal{D}}|$, for each $\widetilde{\mathcal{D}}$ direct successor of $\mathcal{D}$.

Since the enumeration of all DAGs in $\mathcal{S}_q$ is unfeasible in practice, direct sampling from the baseline $p(\mathcal{D})$ can be performed through an acceptance-rejection method. For a given DAG $\mathcal{D}$, let $N(\mathcal{D})$ be the set of all its direct successors, each one obtained by applying an operator in the set $\mathcal{O}_{\mathcal{D}}$ defined above. We first uniformly sample a DAG $\widetilde{\mathcal{D}}$ from $N(\mathcal{D})$, which occurs with probability $q(\widetilde{\mathcal{D}} \,|\, \mathcal{D}) = 1/|N(\mathcal{D})|$, for each $\widetilde{\mathcal{D}} \in N(\mathcal{D})$. Hence, we move to $\widetilde{\mathcal{D}}$ with probability

$$\alpha_{\widetilde{\mathcal{D}}} = \min\left\{1; \frac{p(\widetilde{\mathcal{D}})}{p(\mathcal{D})} \cdot \frac{q(\mathcal{D} \,|\, \widetilde{\mathcal{D}})}{q(\widetilde{\mathcal{D}} \,|\, \mathcal{D})}\right\}.$$

Importantly, to compute $\alpha_{\widetilde{\mathcal{D}}}$ we only need to evaluate the *ratio* of the priors $p(\widetilde{\mathcal{D}})/p(\mathcal{D}) = r$, which avoids the computation of normalizing constants over the space of DAGs; see also Section 3.2 of the main paper. Moreover, the ratio of the two proposal probabilities reduces to $q(\mathcal{D} \,|\, \widetilde{\mathcal{D}})/q(\widetilde{\mathcal{D}} \,|\, \mathcal{D}) = |\mathcal{O}_{\mathcal{D}}|/|\mathcal{O}_{\widetilde{\mathcal{D}}}|$ which requires the enumeration of all the direct successors of $\mathcal{D}$ and $\widetilde{\mathcal{D}}$. While this is feasible with a relatively small computational cost, it was observed empirically that the approximation $q(\mathcal{D} \,|\, \widetilde{\mathcal{D}})/q(\widetilde{\mathcal{D}} \,|\, \mathcal{D}) \approx 1$ does not produce a relevant loss in terms of accuracy.

3. Proofs of Propositions 1 and 2

In this section we provide the proofs of Propositions 1 and 2 on posterior predictive distributions, whose statements are included in Section 4 of our paper.

3.1. Proof of Proposition 1

Proposition 1 For a given cluster k, consider the data matrix $\boldsymbol{X}^{(k)}$ collecting the n_k observations $\{\boldsymbol{x}^{(l)} : \xi_l = k\}$ and an observation $\boldsymbol{x}^{(i)}$. Then, the posterior predictive of $\boldsymbol{x}^{(i)}$ given $\{\boldsymbol{x}^{(l)} : l \neq i, \xi_l = k\}$ under DAG $\mathcal{D}_k$ is

$$p(\boldsymbol{x}^{(i)} \,|\, \{\boldsymbol{x}^{(l)} : l \neq i, \xi_l = k\}, \mathcal{D}_k) = \prod_{j=1}^{q} \left\{ \frac{a/|\mathcal{X}_{\mathrm{fa}(j)}| + {}_k n^{\mathrm{fa}(j)}_{(\tilde{m}_j, \tilde{s}_j)} - \mathbb{1}\{\xi_i = k\}}{a/|\mathcal{X}_{\mathrm{pa}(j)}| + {}_k n^{\mathrm{pa}(j)}_{\tilde{s}_j} - \mathbb{1}\{\xi_i = k\}} \right\} \tag{3}$$

where $\tilde{m}_j = \boldsymbol{x}^{(i)}_j, \tilde{s}_j = \boldsymbol{x}^{(i)}_{\mathrm{pa}(j)}$ and

$${}_k n^{\mathrm{fa}(j)}_{(\tilde{m}_j, \tilde{s}_j)} = \sum_{l:\xi_l = k} \mathbb{1}\{\boldsymbol{x}^{(l)}_{\mathrm{fa}(j)} = (\tilde{m}_j, \tilde{s}_j)\}, \quad {}_k n^{\mathrm{pa}(j)}_{\tilde{s}_j} = \sum_{l:\xi_l = k} \mathbb{1}\{\boldsymbol{x}^{(l)}_{\mathrm{pa}(j)} = \tilde{s}_j\}.$$

Proof. We first write the posterior predictive as the ratio of two marginal likelihoods, defined as in (2),

$$p\big(\boldsymbol{x}^{(i)}\,|\,\{\boldsymbol{x}^{(l)} : l \neq i, \xi_l = k\}, \mathcal{D}_k\big) = \frac{m\big(\boldsymbol{x}^{(i)}, \boldsymbol{X}^{(k)}_{-i}\,|\,\mathcal{D}_k\big)}{m\big(\boldsymbol{X}^{(k)}_{-i}\,|\,\mathcal{D}_k\big)}, \tag{4}$$

where $\boldsymbol{X}^{(k)}_{-i} = \big\{\boldsymbol{x}^{(l)} : l \neq i, \xi_l = k\big\}$. Now notice that DAG $\mathcal{D}_k$ is the same across numerator and denominator and accordingly all terms in (1) depending on $\mathcal{D}_k$ but not on the data, namely the prior normalizing constants, cancel out. Therefore, we obtain

$$p\big(\boldsymbol{x}^{(i)}\,|\,\{\boldsymbol{x}^{(l)} : l \neq i, \xi_l = k\}, \mathcal{D}_k\big) = \prod_{j=1}^{q}\left\{\prod_{s\in\mathcal{X}_{\mathrm{pa}(j)}} \frac{h\big(\boldsymbol{a}_s^{j\,|\,\mathrm{pa}(j)} + {}_k\boldsymbol{n}_s^{\mathrm{fa}(j)}(-i)\big)}{h\big(\boldsymbol{a}_s^{j\,|\,\mathrm{pa}(j)} + {}_k\boldsymbol{n}_s^{\mathrm{fa}(j)}(+i)\big)}\right\},$$

where ${}_k\boldsymbol{n}_s^{\mathrm{fa}(j)}(-i)$ is the table of counts for variable X_j given level $s \in \mathcal{X}_{\mathrm{pa}(j)}$ which is obtained from the data matrix $\boldsymbol{X}^{(k)}_{-i}$, i.e. using all the observations assigned to cluster k excluding $\boldsymbol{x}^{(i)}$ if $\xi_i = k$. Similarly for ${}_k\boldsymbol{n}_s^{\mathrm{fa}(j)}(+i)$, which is obtained from $\boldsymbol{X}^{(k)}_{+i}$, i.e. all observations assigned to cluster k including $\boldsymbol{x}^{(i)}$ if i is not currently assigned to that cluster, that is if $\xi_i \neq k$. Moreover, by writing explicitly the posterior normalizing constants as in (2), we obtain

$$\begin{aligned}
&p\big(\boldsymbol{x}^{(i)}\,|\,\{\boldsymbol{x}^{(l)} : l \neq i, \xi_l = k\}, \mathcal{D}_k\big)\\
&\quad= \prod_{j=1}^{q}\left\{\prod_{s\in\mathcal{X}_{\mathrm{pa}(j)}} \frac{\Gamma\left(a/|\mathcal{X}_{\mathrm{pa}(j)}| + {}_kn_s^{\mathrm{pa}(j)}(-i)\right)}{\Gamma\left(a/|\mathcal{X}_{\mathrm{pa}(j)}| + {}_kn_s^{\mathrm{pa}(j)}(+i)\right)} \prod_{m\in\mathcal{X}_j} \frac{\Gamma\left(a/|\mathcal{X}_{\mathrm{pa}(j)}| + {}_kn_{(m,s)}^{\mathrm{fa}(j)}(+i)\right)}{\Gamma\left(a/|\mathcal{X}_{\mathrm{pa}(j)}| + {}_kn_{(m,s)}^{\mathrm{fa}(j)}(-i)\right)}\right\}.
\end{aligned}$$

Now notice that the counts involved in the previous expression only differ by the inclusion/removal of subject $\boldsymbol{x}^{(i)}$ from the data matrix $\boldsymbol{X}^{(k)}$. As a consequence, all counts in ${}_k\boldsymbol{n}_s^{\mathrm{fa}(j)}(+i)$ and ${}_k\boldsymbol{n}_s^{\mathrm{fa}(j)}(-i)$ are equal, except for those corresponding to the configuration attained by $\boldsymbol{x}^{(i)}$, say $\tilde{s}_j = \boldsymbol{x}^{(i)}_{\mathrm{pa}(j)}$ and $\tilde{m}_j = \boldsymbol{x}^{(i)}_j$; similarly for ${}_k\boldsymbol{n}_s^{\mathrm{pa}(j)}(+i)$ and ${}_k\boldsymbol{n}_s^{\mathrm{pa}(j)}(-i)$. Accordingly, the predictive probability above simplifies to

$$\begin{aligned}
&p\big(\boldsymbol{x}^{(i)}\,|\,\{\boldsymbol{x}^{(l)} : l \neq i, \xi_l = k\}, \mathcal{D}_k\big)\\
&\quad= \prod_{j=1}^{q}\left\{\frac{\Gamma\left(a/|\mathcal{X}_{\mathrm{pa}(j)}| + {}_kn_{\tilde{s}_j}^{\mathrm{pa}(j)}(-i)\right)}{\Gamma\left(a/|\mathcal{X}_{\mathrm{pa}(j)}| + {}_kn_{\tilde{s}_j}^{\mathrm{pa}(j)}(+i)\right)} \cdot \frac{\Gamma\left(a/|\mathcal{X}_{\mathrm{fa}(j)}| + {}_kn_{(\tilde{m}_j,\tilde{s}_j)}^{\mathrm{fa}(j)}(+i)\right)}{\Gamma\left(a/|\mathcal{X}_{\mathrm{fa}(j)}| + {}_kn_{(\tilde{m}_j,\tilde{s}_j)}^{\mathrm{fa}(j)}(-i)\right)}\right\}.
\end{aligned} \tag{5}$$

We can now distinguish two cases: (i) $\xi_i = k$ and (ii) $\xi_i \neq k$.

In case (i), because i is currently assigned to cluster k, we actually need to remove it from $\boldsymbol{X}^{(k)}$. Therefore, we have ${}_kn_{\tilde{s}_j}^{\mathrm{pa}(j)}(-i) = {}_kn_{\tilde{s}_j}^{\mathrm{pa}(j)} - 1$ and similarly ${}_kn_{(\tilde{m}_j,\tilde{s}_j)}^{\mathrm{fa}(j)} = {}_kn_{(\tilde{m}_j,\tilde{s}_j)}^{\mathrm{fa}(j)} - 1$, while ${}_kn_{\tilde{s}_j}^{\mathrm{pa}(j)}(+i) = {}_kn_{\tilde{s}_j}^{\mathrm{pa}(j)}$ and ${}_kn_{(\tilde{m}_j,\tilde{s}_j)}^{\mathrm{fa}(j)}(+i) = {}_kn_{(\tilde{m}_j,\tilde{s}_j)}^{\mathrm{fa}(j)}$.
As a consequence, Equation (5) becomes

$$\begin{aligned}
&p\big(\boldsymbol{x}^{(i)}\,|\,\{\boldsymbol{x}^{(l)} : l \neq i, \xi_l = k\}, \mathcal{D}_k\big)\\
&\quad= \prod_{j=1}^{q}\left\{\frac{\Gamma\left(a/|\mathcal{X}_{\mathrm{pa}(j)}| + {}_kn_{\tilde{s}_j}^{\mathrm{pa}(j)} - 1\right)}{\Gamma\left(a/|\mathcal{X}_{\mathrm{pa}(j)}| + {}_kn_{\tilde{s}_j}^{\mathrm{pa}(j)}\right)} \cdot \frac{\Gamma\left(a/|\mathcal{X}_{\mathrm{fa}(j)}| + {}_kn_{(\tilde{m}_j,\tilde{s}_j)}^{\mathrm{fa}(j)}\right)}{\Gamma\left(a/|\mathcal{X}_{\mathrm{fa}(j)}| + {}_kn_{(\tilde{m}_j,\tilde{s}_j)}^{\mathrm{fa}(j)} - 1\right)}\right\}.
\end{aligned}$$

Finally, by using the property of Gamma functions $\Gamma(c+1) = c\Gamma(c)$, for arbitrary $c > 0$, we obtain

$$p\big(\boldsymbol{x}^{(i)} \,|\, \{\boldsymbol{x}^{(l)} : l \neq i, \xi_l = k\}, \mathcal{D}_k\big) = \prod_{j=1}^{q} \left\{ \frac{\Big(a/|\mathcal{X}_{\mathrm{fa}(j)}| + {}_k n^{\mathrm{fa}(j)}_{(\tilde{m}_j, \tilde{s}_j)} - 1\Big)}{\Big(a/|\mathcal{X}_{\mathrm{pa}(j)}| + {}_k n^{\mathrm{pa}(j)}_{\tilde{s}_j} - 1\Big)} \right\}.$$

In case (ii) instead, because individual i is not in cluster k, we actually need to include it. Therefore, ${}_k n^{\mathrm{pa}(j)}_{\tilde{s}_j}(+i) = {}_k n^{\mathrm{pa}(j)}_{\tilde{s}_j} + 1$ and ${}_k n^{\mathrm{fa}(j)}_{(\tilde{m}_j, \tilde{s}_j)}(+i) = {}_k n^{\mathrm{fa}(j)}_{(\tilde{m}_j, \tilde{s}_j)} + 1$, while ${}_k n^{\mathrm{pa}(j)}_{\tilde{s}_j}(-i) = {}_k n^{\mathrm{pa}(j)}_{\tilde{s}_j}$ and ${}_k n^{\mathrm{fa}(j)}_{(\tilde{m}_j, \tilde{s}_j)}(-i) = {}_k n^{\mathrm{fa}(j)}_{(\tilde{m}_j, \tilde{s}_j)}$. Therefore, by proceeding similarly as in case (i), we obtain

$$p\big(\boldsymbol{x}^{(i)} \,|\, \{\boldsymbol{x}^{(l)} : l \neq i, \xi_l = k\}, \mathcal{D}_k\big) = \prod_{j=1}^{q} \left\{ \frac{\Big(a/|\mathcal{X}_{\mathrm{fa}(j)}| + {}_k n^{\mathrm{fa}(j)}_{(\tilde{m}_j, \tilde{s}_j)}\Big)}{\Big(a/|\mathcal{X}_{\mathrm{pa}(j)}| + {}_k n^{\mathrm{pa}(j)}_{\tilde{s}_j}\Big)} \right\}.$$

Finally, we can combine the results under cases (i) and (ii) by writing

$$p(\boldsymbol{x}^{(i)} \,|\, \{\boldsymbol{x}^{(l)} : l \neq i, \xi_l = k\}, \mathcal{D}_k) = \prod_{j=1}^{q} \left\{ \frac{a/|\mathcal{X}_{\mathrm{fa}(j)}| + {}_k n^{\mathrm{fa}(j)}_{(\tilde{m}_j, \tilde{s}_j)} - \mathbb{1}\{\xi_i = k\}}{a/|\mathcal{X}_{\mathrm{pa}(j)}| + {}_k n^{\mathrm{pa}(j)}_{\tilde{s}_j} - \mathbb{1}\{\xi_i = k\}} \right\} \tag{6}$$

where $\mathbb{1}\{\xi_i = k\} = 1$ if $\xi_i = k$, 0 otherwise, and the expression coincides with the statement of the proposition.

3.2. Proof of Proposition 2

Proposition 2 For a new cluster $k = K+1$, the posterior predictive of $\boldsymbol{x}^{(i)}$ coincides with the marginal likelihood and is given by

$$p(\boldsymbol{x}^{(i)} \,|\, \mathcal{D}_k) = \prod_{j=1}^{q} \frac{1}{|\mathcal{X}_j|}. \tag{7}$$

Proof. Notice that if $k = K+1$ is an empty cluster containing no observations, the posterior predictive coincides with $p(\boldsymbol{x}^{(i)} \,|\, \mathcal{D}_k)$, namely the marginal likelihood of DAG $\mathcal{D}_k$ evaluated at $\boldsymbol{x}^{(i)}$ only. Accordingly, to all configurations that are different from the the one attained by $\boldsymbol{x}^{(i)}$, will be assigned frequencies equal to zero. Therefore, if we let $\tilde{s}_j = \boldsymbol{x}^{(i)}_{\mathrm{pa}(j)}$ and $\tilde{m}_j = \boldsymbol{x}^{(i)}_j$, the general expression of the marginal likelihood in Equation (2) reduces to

$$p(\boldsymbol{x}^{(i)} \,|\, \mathcal{D}_k) = \prod_{j=1}^{q} \left\{ \frac{\Gamma(a/|\mathcal{X}_{\mathrm{pa}(j)}|)}{\Gamma(a/|\mathcal{X}_{\mathrm{pa}(j)}| + n^{\mathrm{pa}(j)}_{\tilde{s}_j})} \cdot \frac{\Gamma(a/|\mathcal{X}_{\mathrm{fa}(j)}| + n^{\mathrm{fa}(j)}_{(\tilde{m}_j, \tilde{s}_j)})}{\Gamma(a/|\mathcal{X}_{\mathrm{fa}(j)}|)} \right\}. \tag{8}$$

Moreover, since the marginal likelihood is based on a sample of size one, $\boldsymbol{x}^{(i)}$, we have $n^{\mathrm{pa}(j)}_{\tilde{s}_j} = 1$ and $n^{\mathrm{fa}(j)}_{(\tilde{m}_j, \tilde{s}_j)} = 1$. Again using the property of Gamma functions for which $\Gamma(c+1) = c\Gamma(c)$ with $c > 0$, Equation (8) reduces to

$$p(\boldsymbol{x}^{(i)} \,|\, \mathcal{D}_k) = \prod_{j=1}^{q} \left\{ \frac{a/|\mathcal{X}_{\mathrm{fa}(j)}|}{a/|\mathcal{X}_{\mathrm{pa}(j)}|} \right\} = \prod_{j=1}^{q} \frac{1}{|\mathcal{X}_j|},$$

which coincides with the statement of the proposition.

4. Simulations for clustering and structure learning

We conduct simulation studies to evaluate the performance of our methodology relative to the tasks of clustering and structure learning, and include comparisons with alternative approaches.

4.1. Scenarios

We fix the number of variables $q = 20$, while consider scenarios differing by the number of clusters $K \in \{2, 4\}$, and sample sizes $n_k \in \{100, 200, 500\}$ that are equal across clusters $k = 1, \ldots, K$. We generate the K DAGs independently, so that the K clusters will differ in general by the dependence structure among variables, and with a probability of edge inclusion $\pi = 0.1$. K categorical datasets $\boldsymbol{X}^{(1)}, \ldots, \boldsymbol{X}^{(K)}$ are built by discretization of latent Gaussian observations as follows. Each Gaussian dataset $\boldsymbol{Z}^{(k)}$ collects n_k independent draws from $\mathcal{N}_q(\boldsymbol{0}, \boldsymbol{\Sigma}_k)$, where $\boldsymbol{\Sigma}_k$ is recovered from the modified Cholesky decomposition $\boldsymbol{\Sigma}_k = \boldsymbol{L}_k^{-\top} \boldsymbol{D}_k \boldsymbol{L}_k^{-1}$. Specifically, $\boldsymbol{D}_k$ is a diagonal matrix collecting node-conditional variances, while $\boldsymbol{L}_k$ has all diagonal entries equal to one and off-diagonal elements different from zero if and only if $u \to v$ is in $\mathcal{D}_k$. We randomly draw the non-null elements of $\boldsymbol{L}_k$ uniformly in $[-2, -1.1] \cup [1.1, 2]$, while we fix $\boldsymbol{D}_k = \boldsymbol{I}_q$. These latent data are then discretized into binary observations as

$$\boldsymbol{X}_{ij}^{(k)} = \begin{cases} 0 & \text{if } \boldsymbol{Z}_{ij}^{(k)} < \gamma_j^{(k)} \\ 1 & \text{if } \boldsymbol{Z}_{ij}^{(k)} \geq \gamma_j^{(k)} \end{cases}$$

where $\gamma_j^{(k)} \in (-\infty, +\infty)$ is a threshold that we generate randomly across variables and clusters. Values of such thresholds have an effect on cluster identification, because they determine the proportions of $0-1$ values in the (marginal) distributions of variables and importantly their balance across clusters. We sample $\gamma_j^{(k)} \sim \text{Unif}(\hat{z}_{j,\alpha}^{(k)}, \hat{z}_{j,1-\alpha}^{(k)})$, where $\hat{z}_{j,\alpha}^{(k)}$ denotes the quantile of order α in the empirical distribution of $Z_j^{(k)}$. As such, the value of α implies differences in the marginal distributions of the q variables across clusters. In particular, when α is close to 0.5 marginal distributions are more similar across groups, with approximately same proportion of $0 - 1$ values; accordingly, cluster identification might be more difficult because differences between clusters are only due to different conditional independencies among variables. To appreciate this effect, we then consider two distinct scenarios: $\alpha = 0.1$, in which marginal distributions are in general different across clusters, and $\alpha = 0.4$, which instead implies marginals to be more similar across clusters. For benchmark methods that do not consider a dependence structure between variables we expect a lower ability in recovering the true clustering when $\alpha = 0.4$. Finally, under each scenario defined by K, n_k and α, we generate a collection of $N = 40$ multiple (K) datasets.

4.2. Clustering

We evaluate the clustering performance of our method (DAG mixture) and compare it with state-of-the-art approaches, specifically the Latent Class Model (LCM) (Goodman, 1974; Linzer and Lewis, 2011) and the *k-modes* algorithm (Huang, 1998). Both methods require a fixed number of clusters K that, to ease the implementation, we set equal to the true value in each scenario. Performances are assessed by comparing the true partition $\boldsymbol{c}$ with any estimated partition $\widehat{\boldsymbol{c}}$ obtained from one of the methods, based on the Variation of Information (VI). Lower values of the metric correspond to better performances. Results are summarized in the boxplots of Figure 1. As it appears, all methods tend to improve as the sample size n_k grows, with our DAG mixture model clearly outperforming all the benchmarks under all scenarios.

4.3. Structure learning

We now assess the ability of our method in recovering the graphical structure underlying each cluster. To this end, we consider the Structural Hamming Distance (SHD), which represents the number of

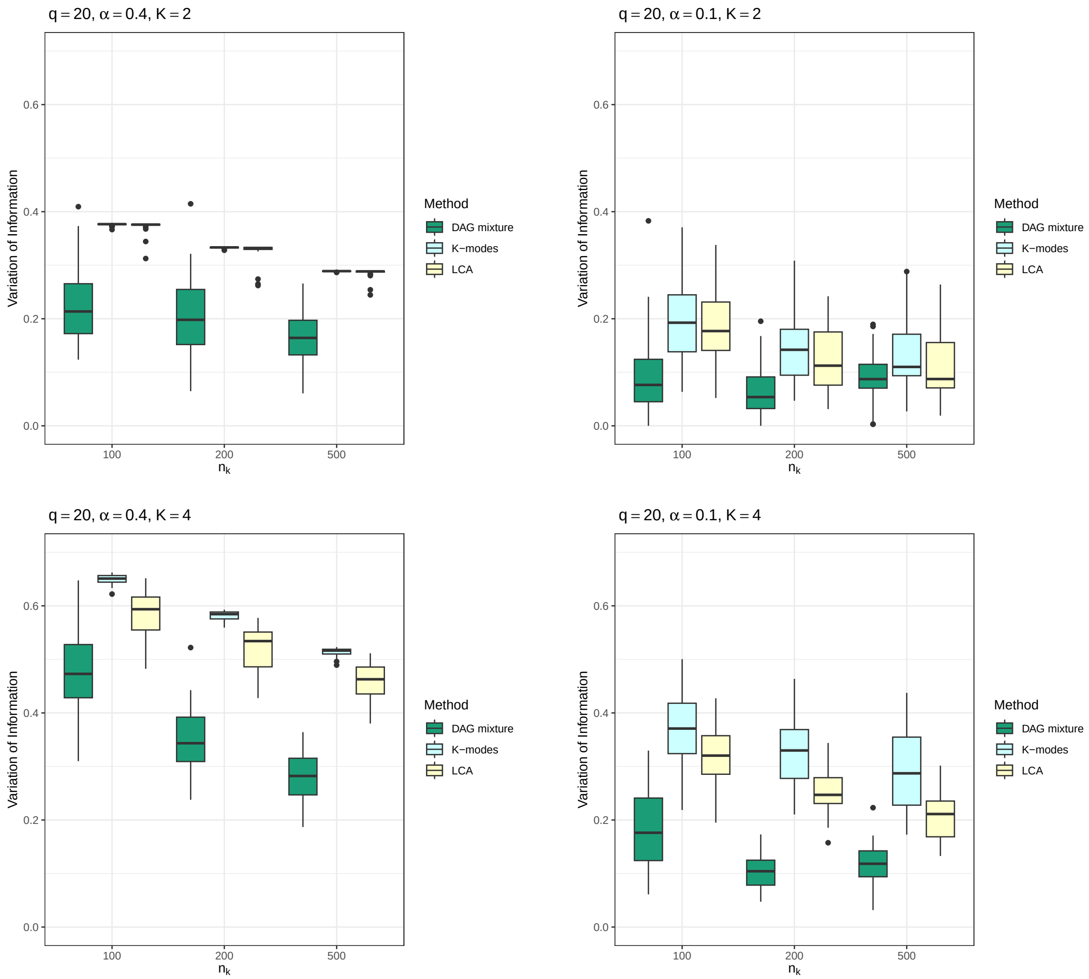

Figure 1 Simulations. Distribution (across 40 replicates) of Variation of Information, for different simulation scenarios characterized by $K \in \{2, 4\}$ and $\alpha \in \{0.1, 0.4\}$. Methods under comparison are: Latent Class Model (LCA), K-modes, and our DP mixture of DAGs (DAG mixture).

modifications (edge insertions, removals or reversals) that are needed to transform one DAG into another. Specifically, we compare each subject-specific estimated DAG $\widehat{\mathcal{D}}_i, i = 1, \ldots, n$ with the true DAG $\mathcal{D}_{\xi_i}$, where ξ_i is the true class-membership. In addition, we include an Oracle version of our method, in which the true clustering is assumed to be known, and a "one-group" naive strategy (No mixture), which instead assigns all subjects to the same cluster and therefore disregards heterogeneity. Results, for each scenario defined by K, α and increasing sample sizes n_k, are summarized in Figure 2. The No mixture strategy, which neglects the clustering structure in the data, performs worse than the other two methods under all scenarios, particularly as the sample size n_k increases. By converse, the Oracle version of our method performs slightly better than our DP mixture method, a behavior which is more evident under scenario $\alpha = 0.4$ where clustering is indeed more challenging. Finally, both DAG Mixture and its Oracle version improve their performance as n_k grows.

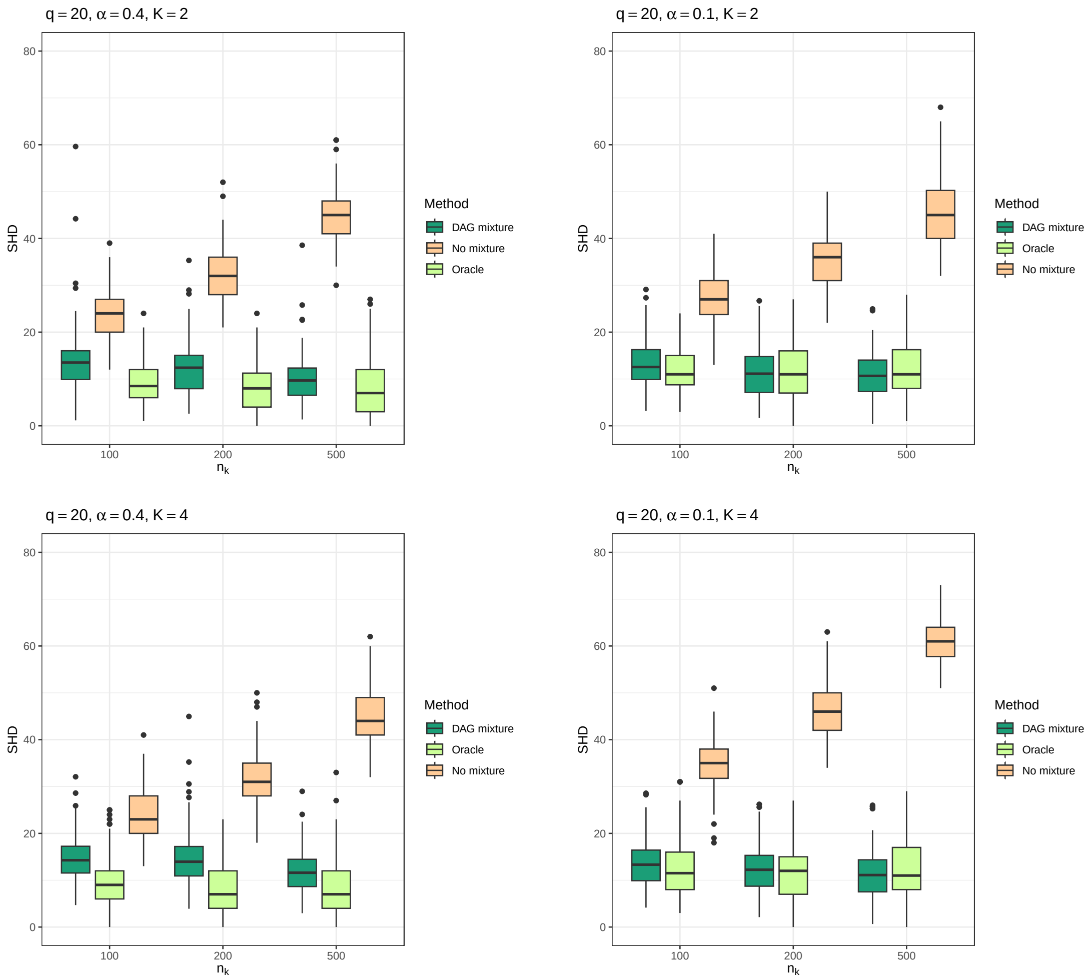

Figure 2 Simulations. Distribution (across 40 replicates) of the SHD between estimated and true DAGs, for different simulation scenarios characterized by $K \in \{2, 4\}$, $\alpha \in \{0.1, 0.4\}$ and increasing sample sizes n_k. Methods under comparison are: No mixture, the Oracle version of our method (Oracle) and our DP mixture of DAGs (DAG mixture).

5. Simulations for causal effect estimation

We conduct simulation studies to evaluate the performance of our method in heterogeneous causal effect estimation. To this end, we consider scenarios with number of variables $q = 10$ and $K = 2$ clusters. Two DAGs, $\mathcal{D}_1$ and $\mathcal{D}_2$ are generated independently according to a parent ordering of the nodes which guarantees DAG identifiability. In such a way, causal effects are also identifiable, which allows to evaluate the effective ability of the methods in causal effect estimation, removing any source of uncertainty which is due to Markov equivalence of DAGs.

Conditionally on each true DAG $\mathcal{D}_k$, we randomly generate the parameter $\boldsymbol{\theta}$ directly from the prior $p(\boldsymbol{\theta})$, Equation (10) of the main text, namely by sampling from the related collection of (conditional) Dirichlet distributions, for fixed value of the hyperparameter $a = 2$. We focus on the causal effects

of nodes $X_h, h \in \{2,9\}$ on $Y = X_1$, whose true values, under each cluster k, are denoted as $\gamma_{h,1}^k$ and recovered through Equation (6). These are summarized in Table 1. Datasets of size n_k are then

	$h=2$	$h=9$
$k=1$	0.000	-0.504
$k=2$	-0.541	0.262

Table 1 Simulations. True causal effects of X_h on $Y = X_1$, for each $h \in \{2,9\}$ and true cluster $k \in \{1,2\}$.

generated from a categorical DAG model based on the sampling distribution (1) in the main text. We repeat the data simulation $N = 40$ times under each scenario corresponding to a sample size $n_k \in \{100, 200, 500\}$.

We implement our method under the same settings of Section 4, to recover subject-specific BMA causal effect estimates $\widehat{\gamma}_i^{\text{BMA}}$; see also Equation (18) of our paper. As a benchmark method for heterogeneous causal inference based on clustering techniques and graphical models, we also consider the following strategy, named LCA-HC. We first apply the Latent Class Analysis (LCA) method (as described in Section 4) to estimate a clustering partition of the data. Next, for each identified cluster, we implement the Hill Climbing (HC) method with the categorical BIC as a score for DAG estimation (Scutari, 2010). Finally, under each DAG, we estimate the DAG parameter $\boldsymbol{\theta}$, through Maximum Likelihood; both the HC method and ML DAG-parameter estimation are provided in the `bnlearn` package; see again (Scutari, 2010). The corresponding causal effect estimates are then obtained by exploiting the relationship between γ and $\boldsymbol{\theta}$ in Equation (6) of our paper. We then recover subject specific causal effects, denoted as $\widehat{\gamma}_i^{\text{ML}}$, which will be uniform across subjects assigned to the same estimated cluster. For each method under comparison, estimated and true causal effects are compared *via* the Absolute Error (AE) distance, which, for given intervened node h, is defined as

$$AE_i = |\widehat{\gamma}_i - \gamma_{\xi_i}|, \tag{9}$$

where $\xi_i \in \{1,2\}$ is the true cluster membership of subject i. As an overall summary, under each simulated dataset, we finally consider the average $AE = \frac{1}{n}\sum_{i=1}^n AE_i$. Results are summarized in Figure 3, which reports the distribution across simulations of the average AE, for each node $h \in \{2,9\}$ (left and right plots respectively), each of the two methods under comparison, and increasing sample sizes $n_k \in \{100, 200, 500\}$. Our method outperforms the alternative approach, with a performance which clearly improves as the sample size grows.

6. Breast cancer data and computational time

In this Section we include descriptive summaries and MCMC diagnostics relative to our application to breast cancer data (Section 6 of the paper). In addition, we discuss the computational time of our MCMC sampler.

6.1. Breast cancer data

6.1.1. Descriptive summaries

Table 2 provides descriptive summary statistics for the analyzed dataset. For each clinical feature, the third column reports the marginal distribution in the full sample, while the next two columns report the corresponding distributions conditionally on each of the two possible administrated treatments (AC = 1 and antiHER2 = 1 respectively); also note that patients could have received either one treatment alone,

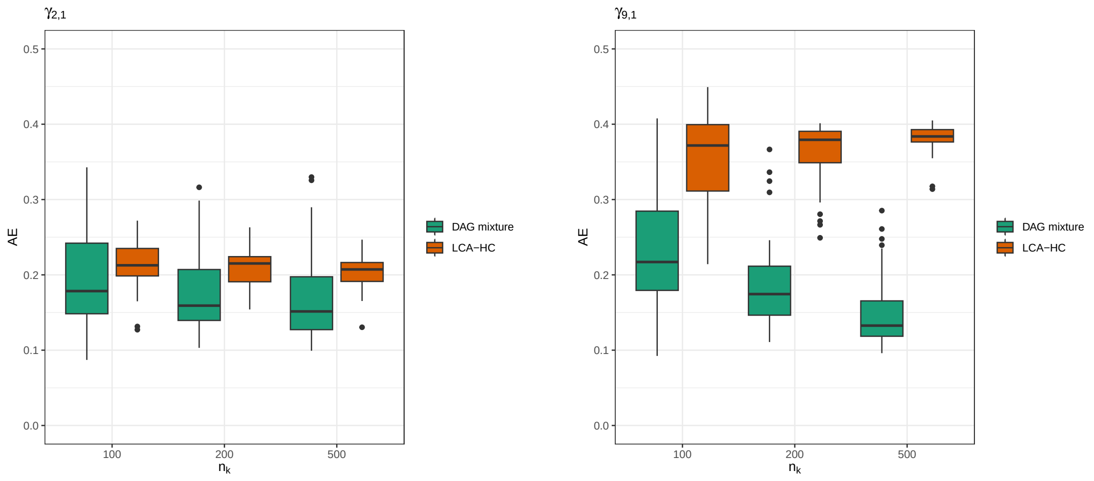

Figure 3 Simulations. Distribution (across 40 replicates) of the Absolute Error between estimated causal effects and true causal effects of X_h on Y, with $h \in \{2, 9\}$. Methods under comparison are: our DAG-mixture model with output a BMA estimate of the causal effect, LCA-HC based on a Maximum Likelihood estimate of the causal effect.

neither treatment, or both treatments, so that the two groups overlap. However, the sample proportions in the treatment groups are broadly comparable with the unconditional proportions observed in the full cohort. In addition, a few levels are associated with small frequencies; their estimation is however regularized through prior information which is gained from the Dirichlet prior on the conditional probability parameters.

6.1.2. MCMC diagnostics

To assess the convergence and mixing of our MCMC algorithm, we rely on the following synthetic summaries and features characterizing the posterior distribution:

- log-marginal likelihood;
- posterior expectation of the number of clusters K;
- entropy of the clustering partition.

The first quantity corresponds to the marginal data distribution, which is obtained after integrating out the DAG-parameters, namely

$$p\left(\boldsymbol{X} \,|\, \{\xi_i\}_{i=1}^n, \{\mathcal{D}_i\}_{i=1}^n\right) = \prod_{k=1}^K \int \left\{ \prod_{i:\xi_i=k} p\left(\boldsymbol{x}^{(i)} \,|\, \boldsymbol{\theta}^\star_{\xi_i}, \mathcal{D}^\star_{\xi_i}\right) \right\} p(\boldsymbol{\theta}^\star_k \,|\, \mathcal{D}^\star_k)\, d\boldsymbol{\theta}^\star_k, \tag{10}$$

where the first term in the integral is the likelihood function for cluster k, while $p(\boldsymbol{\theta}^\star_k \,|\, \mathcal{D}_k)$ is the prior on $\boldsymbol{\theta}_k$ (conditionally on DAG $\mathcal{D}^\star_k$) in the DP baseline measure. Each integral is available in closed-form, so that the marginal likelihood can be computed across MCMC iterations $s = 1, \dots, S$, namely conditionally on current values of DAGs and cluster indicator variables. Results in terms of log-marginal likelihood across MCMC iterations are summarized in the first trace-plot of the figure below.

We then consider the expected number of clusters induced by a DP mixture model. In particular, for given value of the DP concentration parameter α, the distribution induced by the DP prior on the

Table 2 Breast cancer data. Descriptive summary statistics for each categorical variable included in the analysis. For each level, the table reports the marginal sample proportion and the corresponding conditional proportions among patients treated with AC and antiHER2.

**ID**	**Level**	**Marginal (%)**	**AC = 1 (72.6%)**	**antiHER2 = 1 (74.3%)**
CTRCD	0	92.8	90.8	92.0
	1	7.2	9.2	8.0
age	0	27.0	29.4	30.7
	1	52.7	54.6	52.0
	2	20.3	16.0	17.3
heart_rate	0	10.7	10.2	11.0
	1	87.6	87.4	87.3
	2	1.7	2.4	1.7
heart_rhythm	0	99.0	99.3	99.0
	1	1.0	0.7	1.0
LVEF	0	2.0	2.1	1.0
	1	69.5	69.6	72.0
	2	28.5	28.3	27.0
HTA	0	77.7	78.8	81.7
	1	22.3	21.2	18.3
DL	0	81.2	82.3	82.3
	1	18.8	17.7	17.7
DM	0	95.0	95.6	96.3
	1	5.0	4.4	3.7
smoker	0	84.9	83.3	84.7
	1	15.1	16.7	15.3
exsmoker	0	84.7	84.0	87.0
	1	15.3	16.0	13.0
RTprev	0	96.5	97.3	97.3
	1	3.5	2.7	2.7
CIprev	0	99.8	100.0	100.0
	1	0.2	0.0	0.0
ICMprev	0	98.5	98.6	98.7
	1	1.5	1.4	1.3
ARRprev	0	97.0	98.3	97.0
	1	3.0	1.7	3.0
VALVprev	0	98.5	99.3	99.3
	1	1.5	0.7	0.7
cxvalv	0	99.8	99.7	99.7
	1	0.2	0.3	0.3
BMI	0	43.1	45.1	44.0
	1	46.0	44.0	46.0
	2	10.9	10.9	10.0

number of clusters K is such that

$$\mathbb{E}(K \mid \alpha) \approx \alpha \log\Big(\frac{\alpha + n}{\alpha}\Big)\,;$$

see in particular Pitman (2006). Having assumed the prior $\alpha \sim \text{Gamma}(c, d)$, we can recover from our MCMC the approximate posterior distribution of α, from which S samples $\alpha^{(1)}, \ldots, \alpha^{(S)}$ are available, and in turn S MCMC samples for $\mathbb{E}(K \mid \boldsymbol{X})$. Results are summarized in the second trace-plot of the figure, which reports sampled values across MCMC iterations.

Finally, we consider the Shannon entropy among cluster indicators $\xi_1, \ldots, \xi_n$. The latter is defined as

$$H(\xi_1, \ldots, \xi_n) = -\sum_{k=1}^{K} \pi_k \log \pi_k$$

where K is the number of clusters, and $\pi_k = n^{-1} \sum_{i=1}^{n} \mathbb{1}(\xi_i = k)$ is the proportion of observations belonging to cluster k, for $k = 1, \ldots, K$. For each MCMC realization of cluster indicators $\xi_1^{(s)}, \ldots, \xi_n^{(s)}$ we can provide a related value of the index, say $H(\xi_1^{(s)}, \ldots, \xi_n^{(s)})$ and monitor the behavior across MCMC iterations; results are summarized in the third trace-plot of the figure. For all quantities, results suggest a good degree of MCMC mixing. In particular, there are in general no substantial trends, and variability across sampled values is almost uniform around an average value.

6.2. Computational time

Finally, we analyze the computational time required by our MCMC algorithm. Specifically, we investigate the computational time as a function of both the number of nodes $q \in \{10, 20\}$ and total sample size $n \in \{10, \ldots, 2000\}$. For every combination of (q, n) we run $N = 16$ simulations and compute the average time *per* iteration. Results are summarized in the two plots of Figure 5 which report the computational time *per* MCMC iteration for values of $q = 10$ and $q = 20$ (top and bottom plots respectively) and increasing values of the total sample sizes n.

REFERENCES

F. Castelletti, G. Consonni, and M. L. Della Vedova. Joint structure learning and causal effect estimation for categorical graphical models. *Biometrics*, 80(3):ujae067, 2024.

L. A. Goodman. Exploratory latent structure analysis using both identifiable and unidentifiable models. *Biometrika*, 61(2):215 – 231, 1974.

Z. Huang. Extensions to the k-means algorithm for clustering large data sets with categorical values. *Data Min Knowl Discov*, 2:283 – 304, 1998.

D. A. Linzer and J. B. Lewis. poLCA: an R package for polytomous variable latent class analysis. *J Stat Softw*, 42(10):1 – 29, 2011.

J. Pitman. *Combinatorial Stochastic Processes*, volume 1875 of *Lecture Notes in Mathematics*. Springer, 2006.

M. Scutari. Learning Bayesian networks with the bnlearn R package. *J Stat Softw*, 35(3):1 – 22, 2010.

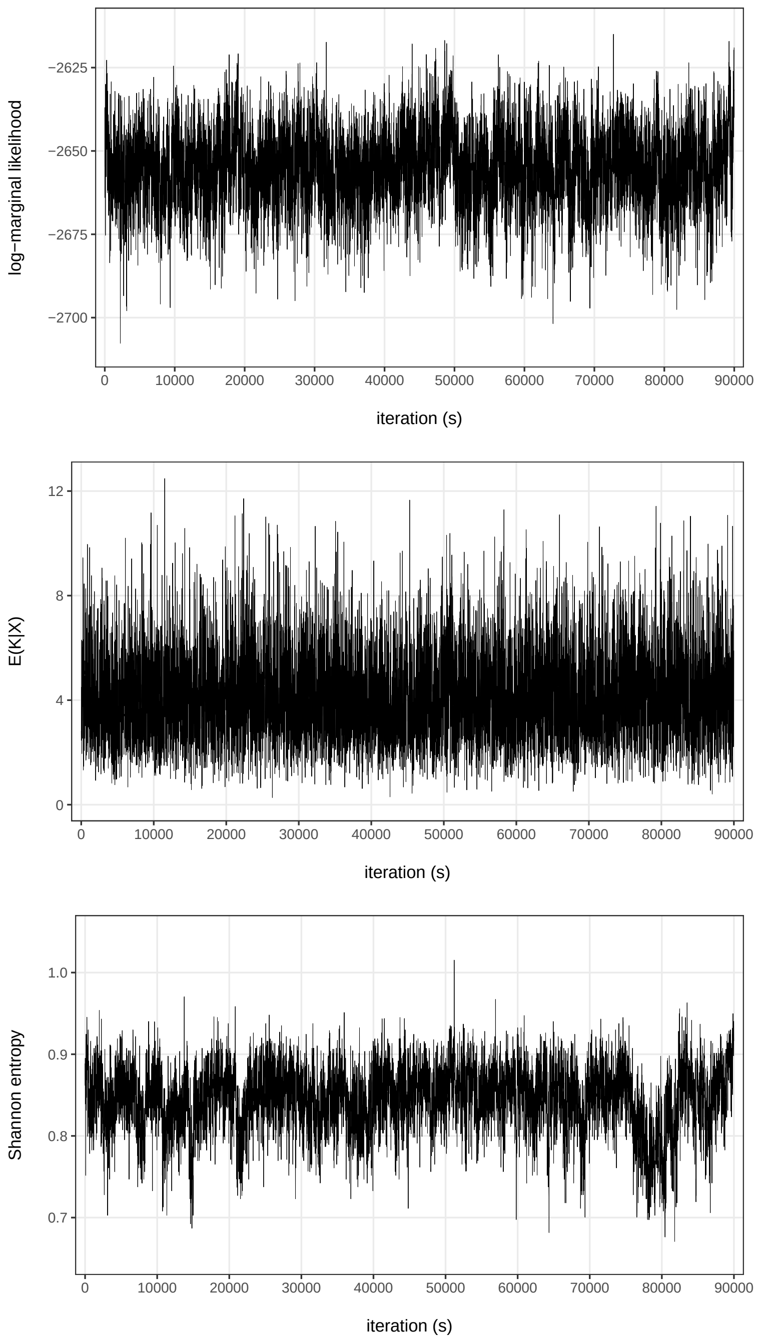

Figure 4 Breast cancer data. From upper to lower panels, trace-plot of log-marginal likelihood, posterior expectation of the number of clusters and Shannon entropy, computed across MCMC iterations.

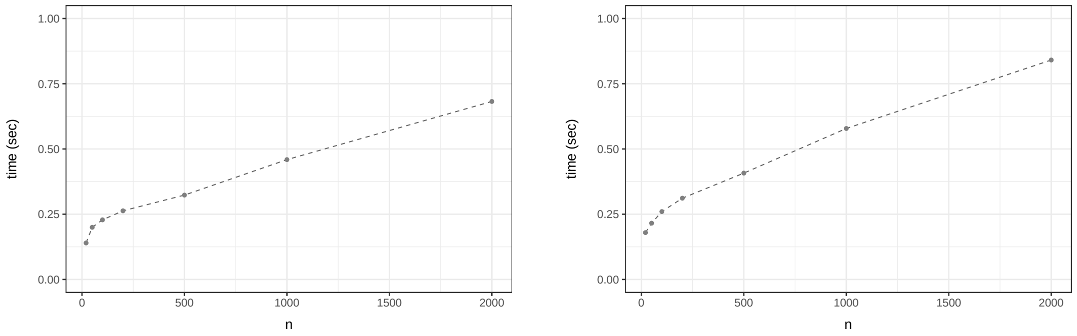

Figure 5 Average computational time (in seconds) of our algorithm *per* MCMC iteration. The two plots refer to fixed number of variables $q = 10$ (top) and $q = 20$ (bottom), and increasing sample size n.
